# The *bs5* allele of the susceptibility gene *Bs5* of pepper (*Capsicum annuum* L.) encoding a natural deletion variant of a CYSTM protein conditions resistance to bacterial spot disease caused by *Xanthomonas* species

**DOI:** 10.1007/s00122-023-04340-y

**Published:** 2023-03-21

**Authors:** Zoltán Szabó, Márta Balogh, Ágota Domonkos, Márta Csányi, Péter Kaló, György B. Kiss

**Affiliations:** 1 Institute of Genetics and Biotechnology, Hungarian University of Agriculture and Life Sciences, Szent-Györgyi A. U. 4., 2100 Gödöllő, Hungary; 2 AMBIS Biotechnology Research and Development Ltd., Budapest, Hungary; 3 Institute of Plant Biology, Biological Research Center, Eötvös Lóránd Research Network, Szeged, Hungary

## Abstract

**Key message:**

The *bs5* resistance gene against bacterial spot was identified by map-based cloning.

**Abstract:**

The recessive *bs5* gene of pepper (*Capsicum annuum* L.) conditions a non-hypersensitive resistance trait, characterized by a slightly swollen, pale green, photosynthetically active leaf tissue, following *Xanthomonas euvesicatoria* infection. The isolation of the *bs5* gene by map-based cloning revealed that the bs5 protein was shorter by 2 amino acids as compared to the wild type Bs5 protein. The natural 2 amino acid deletion occurred in the cysteine-rich transmembrane domain of the tail-anchored (TA) protein, Ca_CYSTM1. The protein products of the wild type *Bs5* and mutant *bs5* genes were shown to be located in the cell membrane, indicating an unknown function in this membrane compartment. Successful infection of the *Bs5* pepper lines was abolished by the 6 bp deletion in the TM encoding domain of the Ca_*CYSTM1* gene in *bs5* homozygotes, suggesting, that the resulting resistance might be explained by the lack of entry of the *Xanthomonas* specific effector molecules into the plant cells.

**Supplementary Information:**

The online version contains supplementary material available at 10.1007/s00122-023-04340-y.

## Introduction

Plants are regularly invaded by beneficial or pathogenic micro-organisms, mainly bacteria and fungi (Wille et al. 2019). Endosymbiotic *Rhizobia*, endomycorrhizal fungi, endophytic, epiphytic and rhizospheric microbes that live inside or outside their host plants all form beneficial associations (Afzal et al. 2019). On the other hand, pathogenic micro-organisms establish harmful interactions which are detrimental to the host plant except when the plant is resistant against the invader. Resistant plants have evolved defense mechanisms which are related to animal innate immunity, but plants have an arsenal of diverse recognition mechanisms as opposed to the animal adaptive immune system (Dodds and Rathjen 2010).

The plant immune system possesses two strategies to detect and fight against pathogens. At first, plants evolved an external receptor system in which pattern recognition receptors (PRRs) can recognize and bind conserved microbial elicitor molecules, called microbial-associated molecular patterns (MAMPs) or pathogen-associated molecular patterns (PAMPs). Stimulation of PRRs by elicitors leads to MAMP-triggered immunity (MTI) or PAMP-triggered immunity (PTI). MTI/PTI is considered to be the primary layer of inducible defense (basal resistance) against pathogenic intruders (Dixon et al. 2000; Jones and Dangl 2006). The detection of certain PAMPs/MAMPs can induce a hypersensitive response (HR), of programmed cell death, at the point of pathogen intrusion (Klement et al. 1964; Flor 1971; Balint-Kurti 2019), although, at present, most PAMPs/MAMPs thus far identified do not induce cell death following perception (Ingle et al. 2006). PTI is more commonly associated with induction of the production of a range of antimicrobial compounds and cell wall thickening, known as callose depositions, in the vicinity of the detected PAMP (Ingle et al. 2006).

Plants also evolved an intracellular receptor system, in which resistance proteins (R proteins) recognize, directly or indirectly, pathogen virulence molecules called effectors (protein products of avirulence genes; Keen 1990; Van der Biezen and Jones 1998). The resulting complex induces effector-triggered immunity (ETI) events that lead to rapid defense response (Jones and Dangl 2006; Boller and Felix 2009; Dodds and Rathjen 2010; Dolatabadian 2020; Harris et al 2020; Wang et al 2022). In contrast to MTI/PTI, in the large majority of cases, ETI is associated with HR (Balint-Kurti 2019). HR is the result of an interaction between a dominant resistance gene (R gene) in the host with a dominant corresponding avirulence gene (Avr gene) in the pathogen. This interaction is referred to as the gene-for-gene relationship, a concept discovered by H. H. Flor and elaborated by other colleagues (Klement et al. 1964; Flor 1971; Higgins et al. 1998; Heath 2000; Balint-Kurti 2019). Resistance is conferred only if both R-gene, present in either homo- or heterozygous genetic configuration, and the corresponding Avr gene are present in the same interaction. Many R-gene and Avr genes have been cloned (Kourelis and van der Hoorn 2018; Garcia-Ruiz et al. 2021; Calle García et al. 2022).

PTI/MTI is generally effective against non-adapted pathogens in a phenomenon called non-host resistance, whereas ETI is active against adapted pathogens. However, these relationships are not exclusive and depend on the elicitor molecules present in each infection. Successful pathogens are able to suppress PTI responses and thereby multiply and cause disease (Heath 2000; Panstruga and Moscou 2020).

Plants are vulnerable to pathogenic microbes when the pathogen produces a virulence factor and the plant can not develop a resistance response but possess dominant susceptibility genes (S genes). In this compatible interaction plants become seriously diseased resulting in an abnormal physiological process that disrupts the plant's normal structure, growth, function, or other activities. Recessive S gene alleles, conferring resistance, have been identified following mutagenesis or as natural variants (e.g., barley *mlo*, *xa13* and *eIF4*, respectively, conferring resistance to powdery mildew, *Xanthomonas* (*X*) bacteria, and potyvirus; Yuan et al. 2009; Kusch and Panstruga 2017; Schmitt-Keichinger 2019). Since recessive S gene alleles conditioning resistance are more durable than major resistance (R) genes (Stall et al. 2009; Palloix et al. 2009), they are more valuable resources in disease resistant crop breeding and that is the case for pepper breeding (Garcia-Ruiz et al. 2021).

Pepper, *Capsicum annuum* L. (*Ca*) and tomato, *Solanum lycopersicum* L. plantations around the world are under continuous threat by the bacterial pathogen, *Xanthomonas euvesicatoria (Xe),* the causal agent of bacterial spot disease (Stall et al. 2009). An epidemic of bacterial spot disease can cause significant loss in pepper and tomato production when elevated temperature combines with high humidity. To fight against this deleterious pathogen in pepper, dominant (*Bs1*, *Bs2*, *Bs3, Bs4C, Bs7*) and recessive (*bs5*, *bs6, bs8*) resistance genes have been identified in wild type pepper species (Ronald and Staskawicz 1988; Jones et al. 2002; Römer et al. 2007; Tai et al. 1999; Potnis et al. 2012; Strauß et al. 2012; Sharma et al. 2022) and introgressed into cultivars (Vallejos et al. 2010). Unfortunately, there is no durable natural resistance determinant against *Xe* in tomato available for breeding (Stall et al. 2009). Dominant *Bs1*, *Bs2*, *Bs3, Bs4C* and *Bs7* genes confer unstable resistance because new races of *Xe* breakdown the resistance trait (Stall et al. 2009). Nevertheless, these dominant genes have been cloned and characterized thoroughly; however, the recessive genes *bs5*, *bs6* and *bs8* remained in obscurity, though the investigation of the molecular basis of recessive genes is relevant in understanding the molecular basis of virulence (Garcia-Ruiz et al. 2021) and the infection process leading to cell and tissue necrosis after infection. In this paper, we describe the map-based cloning and characterization of the *bs5* gene and its protein product conditioning recessive resistance trait against *Xe* in pepper*.*

## Materials and methods

### Bacterium and plant material used in this study

For the determination of disease symptoms, pepper plants were inoculated by pathogenic *Xe* strain *78*, (abbreviated as Xe78) obtained from the microbial collection of Corvinus University of Budapest, Hungary, kindly provided by Laszlo Palkovics. Using specific primer pairs in a PCR amplification experiment, it was demonstrated that Xe78 does not carry genes for *avrBS1*, *avrBS2*, *avrBS3* and *avrBS4.* In addition, implementing a standard identification protocol using differential hosts Xe78 could be classified as Race 10 (Kurowski et al. 2010). *Xe* species were previously designated as *X. campestris* pv. *vesicatoria* (Jones et al. 2006). *X. perforans* (strain NCPPB No.4321) was kindly provided by Jeffrey B. Jones, University of Florida, Gainesville, Florida, USA. *X. gardneri* (kindly provided by Dr. Aleksa Obradovic, University of Belgrade). The following plant materials were used in this study: (i) *Xe* susceptible (Xe^S^) material: *Ca* cv. Early CalWonder (ECW) was kindly provided by Jeffrey B. Jones, University of Florida, Gainesville, Florida, USA. (Jones et al. 2002); *Ca* cv. Feherozon (CaFo), a commertially available Hungarian „Sweet Bell pepper” cultivar (https://www.kertimag.hu/bogyosok/feherozon.html). (ii) Xe resistant (Xe^R^) material: *Ca* cv. 50R (ECW50R) was kindly provided by Jeffrey B. Jones, University of Florida, Gainesville, Florida, USA. (Vallejos et al. 2010); *Ca* var. T1 (CaT1), a single seed plant from accession no. PI163192 of AVRDC—The World Vegetable Center Gene Bank, Shanhua, Taiwan (http://avrdc.org/); *Ca* var. DH99-269 (CaDH269) was kindly provided by G. Csilléry, Budakert Ltd, Budapest, Hungary (Szarka 2008; Csillery et al. 2004); *Ca* cv Global (CaGl) is a commercially available Hungarian cultivar, https://sites.google.com/site/fpkutato/fajtaink/cseresznyepaprika-fajtk). The genotypes of the above plants are given in Fig. S1.

### Plant growth conditions and crossing

Before use, pepper seeds were kept at 4 °C for one to two weeks for vernalization, then sown, geminated and grown under greenhouse conditions in 4 inch pots containing “Florimo®” general flower soil (http://www.florimo.hu/termekeink/viragfoldek/) at 25 °C for 16/8 h light/dark period until flowering. To generate F1 hybrids, maternal flowers were emasculated and manually crossed with pollen from the paternal parent. F2 and F3 seeds were collected from self-pollinated plants.

### *Xe* infection and evaluation of disease symptoms

**Fig. 1:**
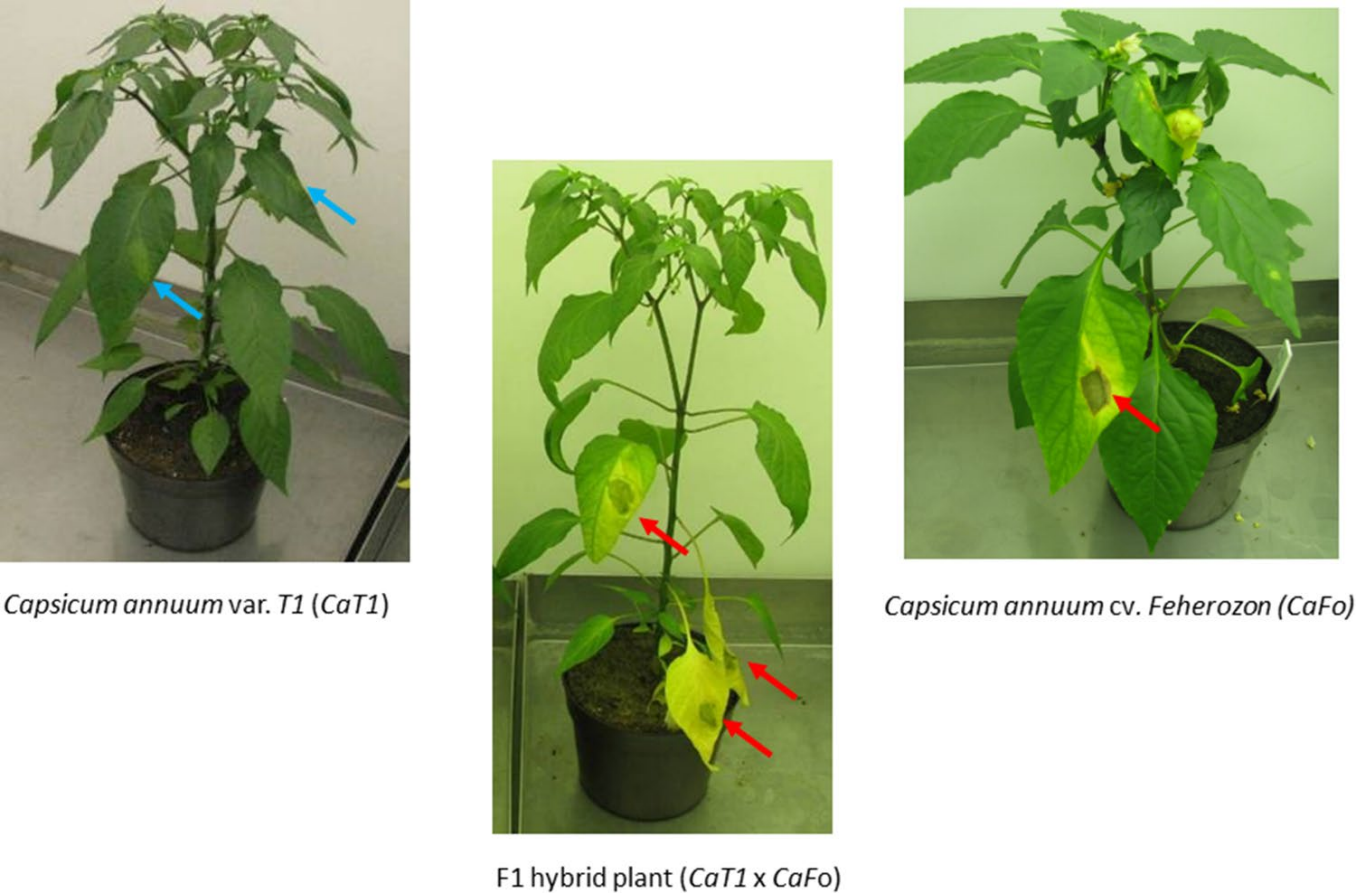
Disease symptoms of leaves following infection with Xe78. Leaf phenotype of parents (CaT1, CaFo) and F1 (CaT1 x CaFo) hybrid plants after 6 days post inoculation with Xe78. Blue arrows show resistant, while red arrows show the susceptible leaf phenotype. The inoculation procedure and phenotypes of the leaves is described in “Materials and methods”

For the determination of the Xe^R^/Xe^S^ phenotype of individual plants, leaf infections with Xe78 bacteria were performed. Bacteria were grown in Nutrient Broth (Sigma-Aldrich Co.) at 28 °C until stationary phase, pelleted by centrifugation and suspended in sterile tap water at a concentration of 10^5^ CFU/mL. Inoculation was done with 1–2 × 10^4^ Xe78 bacteria per leaf. The abaxial side of young leaves (8-leaf stage) was infiltrated into the intercellular space of the leaves with a syringe fitted to a rubber tube. Infected plants were grown under greenhouse conditions as described above. The evaluation of the disease symptoms was as follows: *Xe* resistant plant leaves showed pale green discoloration, healthy, but swollen tissue (Fig. 1) following 6 days after inoculation (DAI) and in later stage as well (Szarka and Csilléry 1995; Szarka et al. 2006; Szarka 2008). Abscission of the leaves was not observed. *Xe* susceptible plant leaves exhibited yellowing, watersoaking after 4–6 DAI, complete necrosis after 6–12 DAI and abscission of the infected leaves after two weeks.

### Allelism testing for *bs5* using reference pepper line

To ascertain whether or not the Xe^R^ plants were in fact carrying a recessive *bs5* allele, allelism tests for *bs5* were performed using ECW50R as reference line. In ECW50R Xe^R^ is conditioned by a single, homozygous recessive gene *bs5* (Vallejos et al. 2010). To this end, ECW50R as paternal parent was crossed with Xe^R^ CaT1, CaGl, CaDH269, respectively, as maternal parents. F1 seeds were germinated, grown and tested for Xe^R^ after inoculation with Xe78 bacteria. All F1 progenies were *Xe* resistant, demonstrating that the recessive gene controlling Xe^R^ in these plants is allelic to *bs5*. Genotyping of the *bs5* allele in the plant used in this study is shown in Fig. S1.

### Construction of pepper BAC library and chromosome walking

A Bacterial Artificial Chromosome (BAC) library was constructed from the *Xe*^R^ pepper line CaDH269*.* This line developed the same resistance symptoms after Xe78 infection as described above for ECW50R and CaT1. In addition, the allelism test and the phenotype of the self-pollinated progeny demonstrated that this plant also carried a *bs5* allele in homozygous configuration. Young leaves from CaDH269 were collected and a BAC library was constructed by BIO SandT Inc. (Montreal, Canada). The bacterial suspension of the BAC library was plated and single colonies were transferred to the cells of 96-well microtiter plates containing 100 μl Nutrient Broth (Sigma-Aldrich Co.) per cell. In total of 380,000 clones were collected and the microtiter plates were arranged in three dimensional array (2 × 3 × 24 plates of 96 wells = 13,824) of which 72 pools were collected (each pool contained 24 × 24 = 576 clones). In this way multiplex PCR could be used to identify single BAC clones with just 72 PCR amplifications and agarose gel-electrophoresis. During chromosomal walking, BAC end specific markers were either used: (i) to isolate overlapping novel BAC clones, (ii) to re-confirm overlapping relationship with the previously identified BAC clones (e.g., primer pairs M_50b4-40 were used to verify the overlap with BAC-326d1, while primer pairs M_50b4-OP were used to identify BAC-137d8, etc.), or (iii) to verify the map position of the *bs5* gene (to avoid miss-orientation).

### Total DNA isolation, PCR amplification and electrophoresis of amplicons

Total DNA was isolated from approximately 100 mg young leaf tissue using the ZenoGene40 Plant DNA Isolation Kit (ZENON Biotechnology Ltd, Szeged, Hungary) according to the supplier’s instructions. PCR amplification was performed using standard conditions in Taq Buffer (ZENON Biotechnology Ltd, Szeged, Hungary) in the presence of Taq DNA polymerase (Dupla-Taq™, ZENON Biotechnology Ltd, Szeged, Hungary), 0.4 μM primers, 1.5 mM MgCl_2_ and 5–10 ng total DNA as template. PCR cycles were carried out, following an initial denaturation step for 3 min at 94 °C, at 94 °C for 1 min, annealing at 45–68 °C (depending on the primer annealing temperature) for 1 min, extension at 72 °C for 1 min over 35 cycles and terminated with 7 min incubation at 72 °C. PCR amplification products were separated in 1–3% agarose gels depending on the expected length of the PCR fragment in 1 × Tris acetate/EDTA buffer. Fragments undistinguishable by length in the gel were subjected to SSCP (Single Strand Conformation Polymorphism; Orita et al. 1989) analysis. Buffer, gels and staining were prepared and performed by standard procedures. Primers used throughout this study are listed in Table S1. Primer names have a “Pr_” prefix and an “F” or “R” suffix. Genetic markers are referred to as the name of the primers (without “F/R” suffix) having an “M_” prefix.

### Preparation of RNA, reverse transcription and transcriptome analysis by RNA-Seq

Young leaves from sensitive and resistant pepper plants were ground in liquid nitrogen then RNA was extracted using the SV Total RNA isolation kit (Promega Corporation, Madison, USA). To reduce the genomic DNA contamination, on-column DNase treatment was carried out. The purification was accomplished according to the manufacturer's instructions. Integrity of the RNA was checked by the Agilent 2100 Bioanalyzer system (Agilent Santa Clara, CA, USA). For the generation of constructs of *Bs5* and *bs5*, cDNA was synthesized according to the supplier’s instructions using the Maxima Reverse Transcriptase (Thermo Fisher Scientific, Waltham, MA).

Before library preparation for total transcriptome analysis, ribosomal RNAs were removed using the Ribo-Zero rRNA removal kit (Epicentre, Madison, USA). Library preparation and RNA sequencing (RNA-Seq) were performed by using the dedicated kits and the total RNA sequencing was carried out using the SOLiD4 sequencer (Life Technologies, Carlsbad, CA, USA) by the Seqomics Ltd. (Mórahalom, Hungary).

### Generating constructs

For reverse complementation experiment, a 3 kb long genomic region with 1 kb native promoter and 440 bps terminator region of *Bs5* were amplified using CaFo genomic DNA as template with specific oligonucleotides extended with the sequences of PstI restriction sites at their 5′ ends (Fig. S2). PstI digested PCR fragments were inserted into PstI site of pCAMBIA1303 binary vector. Hygromycin B phosphotransferase gene was exchanged with the aminoglycoside phosphotransferase (NeoR) gene using *Xho*I restriction sites for generating the same clones with kanamycin resistance.

For GFP-Bs5-fusion constructs, *Bs5* coding sequences were amplified from pepper cDNA using Bs5 specific primers containing NheI and PmlI restriction endonuclease sites (Fig. S3). Template cDNAs were prepared from total RNA of *Bs5* sensitive CaFo and *bs5* resistant CaT1 plants using RevertAid Premium Reverse Transcriptase (Fermentas, Lithuania) with oligo dT20 primer according to the manufacturer's protocol. The amplified *Bs5* and *bs5* cDNA fragments were cloned into pGEMT Easy vector (Promega Corporation, Madison, USA) and then the NcoI-NheI fragment from pCAMBIA1302 containing the mgfp5 gene was inserted in front of *Bs5* variants. NcoI and blunt-ended SalI (filled with Klenow enzyme) fragments containing the GFP-Bs5 and GFP-bs5 fusion proteins were inserted into NcoI and blunt ended BstEII site of the pCAMBIA1302 binary vector.

### Transient gene expression in *N. benthamiana* epidermal cells and microscopy analysis

The coding region of the CaFo-*Bs5* and CaT1*-bs5* cDNAs were cloned at the C-terminal end of the green fluorescent protein (GFP) of pCambia-1302 (see Fig S3). The resulting plasmids pCambia-1302*-*GFP*-Bs5* and pCambia-1302-GFP-*bs5* were introduced from *E. coli* into *Agrobacterium tumefaciens* strain C58 by tri-parental mating and were infiltrated into young *Nicotiana benthamiana* leaves along an *E. coli* strain expressing P19 RNA silencing suppressor. After 48 h, protoplasts were prepared from the infected area using the protoplast isolation method (Nagy and Maliga 1976) and the subcellular localization of GFP fluorescent signal was investigated by Zeiss (Zeiss LSM 510 META) and Olympus (Olympus Fluoview FV1000 Confocal laser scanning microscope) confocal microscopes.

### Transformation of pepper plants

The Hungarian pepper cultivar CaGI carrying the recessive *bs5* gene in homozygous configuration was used in transformation experiments to reverse-complement the mutation in the *bs5* genes (the *bs5*, but not the *Bs5* allele was present in CaGl as demonstrated by allelism test and by PCR based genotyping Fig. S1). In these reverse-complementation experiments transgenic plants carry an extra copy of the wild type *Bs5* gene together with the two copies of the resident *bs5* alleles. The ectopic expression of the *Bs5* gene in the transgenic plants will supply wild type Bs5 functions consequently it is expected that the resistance trait of CaGl will be reversed to susceptibility since *Bs5* gene is dominant over *bs5* (see above, the genetics of this trait show, that F1 plants with *Bs5*/*bs5* genetic configurations are susceptible to Xe78). To this end, CaGl leaves (8 leaf state) were treated by *At ShooterGRif*^*R*^ [pCambia-1303(*Hyg*^R^)-Bs5]*, At ShooterGRif*^*R*^ [pCambia-1303(Neo^R^)-Bs5] and *At ShooterGRif*^*R*^ (pCambia-1303) as a control according to the transformation protocol described by Mihalka et al. (2003). For structural and functional map of plasmid constructs (see Fig. S2).

### Expression of the *Bs5*, *bs5* and *Ca_CYSTM2* genes

Expression of *Bs5*, *bs5*, *Ca_CYSTM2* and *WD40* genes were investigated by whole transcriptome shotgun sequencing (RNA Seq, using SOLiD™ System, http://www.lifetechnologies.com) of RNA isolated from bulked leaf tissue of 8 resistant and 8 susceptible plants of CaFoT1-F2 plants (the same ones used for mapping, see “Materials and methods”). Reads were aligned to the corresponding TC-s. RNA was isolated form *Xe* resistant and susceptible leaf samples at 0 (samples 53 and 55) and 24 h (samples 54 and 56) post infection, respectively. The number of reads was: 20,365,803; 42,128,939; 27,750,667 and 25,427,924 for samples 53, 54, 55 and 56, respectively. There was at least tenfold difference in expression of *Bs5* and *Ca_CYSTM2* in leaves (Z. Szabó unpublished observations). In addition to RNA Seq, EST database of NCBI was also used to extract EST sequences from different tissues.

## Results

### Selecting *Xe* resistant pepper plant and allelism testing for *bs5*

In a search for durable Xe^R^ pepper plants, several seed accessions from the pepper seed stock collection of The World Vegetable Center Genebank (http://avrdc.org/) were screened in pathogeniticity tests. A single pepper plant, denoted CaT1 exhibited robust resistance following inoculation with Xe78. Inoculation with Xe78 resulted in typical water-soaked lesions on the leaves of Xe^S^ plants but Xe^R^ pepper leaves showed only slight leaf changes (Fig. 1). In addition, CaT1 plants were also resistant to *X. perforans*, but were susceptible to *X. gardneri* (data not shown, see also Sharma et al. 2022). The resistant leaf phenotype of the *Xe* infected CaT1 plants resembled that of ECW50R (Vallejos et al. 2010), CaDH269 (Csillery et al. 2004; Szarka et al. 2006) and CaGl (https://sites.google.com/site/fpkutato/fajtaink/cseresznyepaprika-fajtk).

To ascertain whether the above plants were carrying *bs5* allele in homozygous configuration, F1 allelism tests were performed using ECW50R, a reference plant (a *bs5/bs5* homozygote, Jones et al. 2002; Vallejos et al. 2010) as paternal parent, and crossed with CaT1, CaDH269, and CaGl plants, respectively. F1 progeny were tested for *Xe* phenotype after inoculation with Xe78 bacteria and – as a result—all F1 plants were Xe^R^, demonstrating that the recessive gene controlling *Xe *resistance in CaT1, CaDH269, and CaGl, respectively*,* were allelic to *bs5*. Moreover, the homozygosity of plants CaT1, CaDH269, CaGl and ECW50R were further confirmed for the *bs5* locus afterward (Fig. S1) using a *bs5-*specific molecular marker, M_bs5g developed based on the cloned *bs5* gene (see below).

### Genetic mapping of the *bs5* gene

In order to identify the *bs5* gene, map-based cloning was initiated. To establish F2 segregation populations, F1 plants were generated by crossing the Xe^R^ CaT1 plant (**♀**) with the Xe^S^ CaFo(♂) and F1 progenies—a total of 46 individuals—were tested for disease symptoms after inoculation of the leaves with Xe78 bacteria*.* F1 plants and the paternal parent (CaFo) showed the characteristic Xe^S^ symptoms of bacterial spot disease (water-soaked lesions, complete necrosis and abscission of infected leaves) while maternal parent (CaT1) was Xe^R^ (Fig. 1). Based on these results, we concluded that: (i) similarly to the former observations, the susceptibility to Xe78, conditioned by the wild type *Bs5* allele, was a dominant trait over the resistant *bs5* allele; (ii) both *bs5* and *Bs5* were in homozygous configuration in the parental lines; (iii) the F1 plants were derived from crossing and not from self-pollination because no F1 progeny inherited the Xe^R^ (maternal) phenotype. The hybrid nature of the F1 plants was also confirmed with the M_472g4_OP molecular marker (Fig. S4). These results were in agreement with the former findings that the resistance to bacterial spot disease was conditioned by the recessive gene *bs5* (Jones et al. 2002). To identify the chromosomal location of the *bs5* gene, F2 individuals, derived from 46 self-mated F1 plants and termed collectively CaT1Fo F2, were used for genetic mapping*.* A total of 4662 F2 plants were scored for disease symptoms of which 3516 and 1146 were Xe^S^ and Xe^R^, respectively. These figures did not deviate significantly from the expected 3:1 ratio (χ^2^ = 0.44, p = 0.51), therefore we confirmed the hypothesis that the Xe^R^ trait is conditioned by a single recessive gene, *bs5* in this diploid pepper population.

**Fig. 2:**
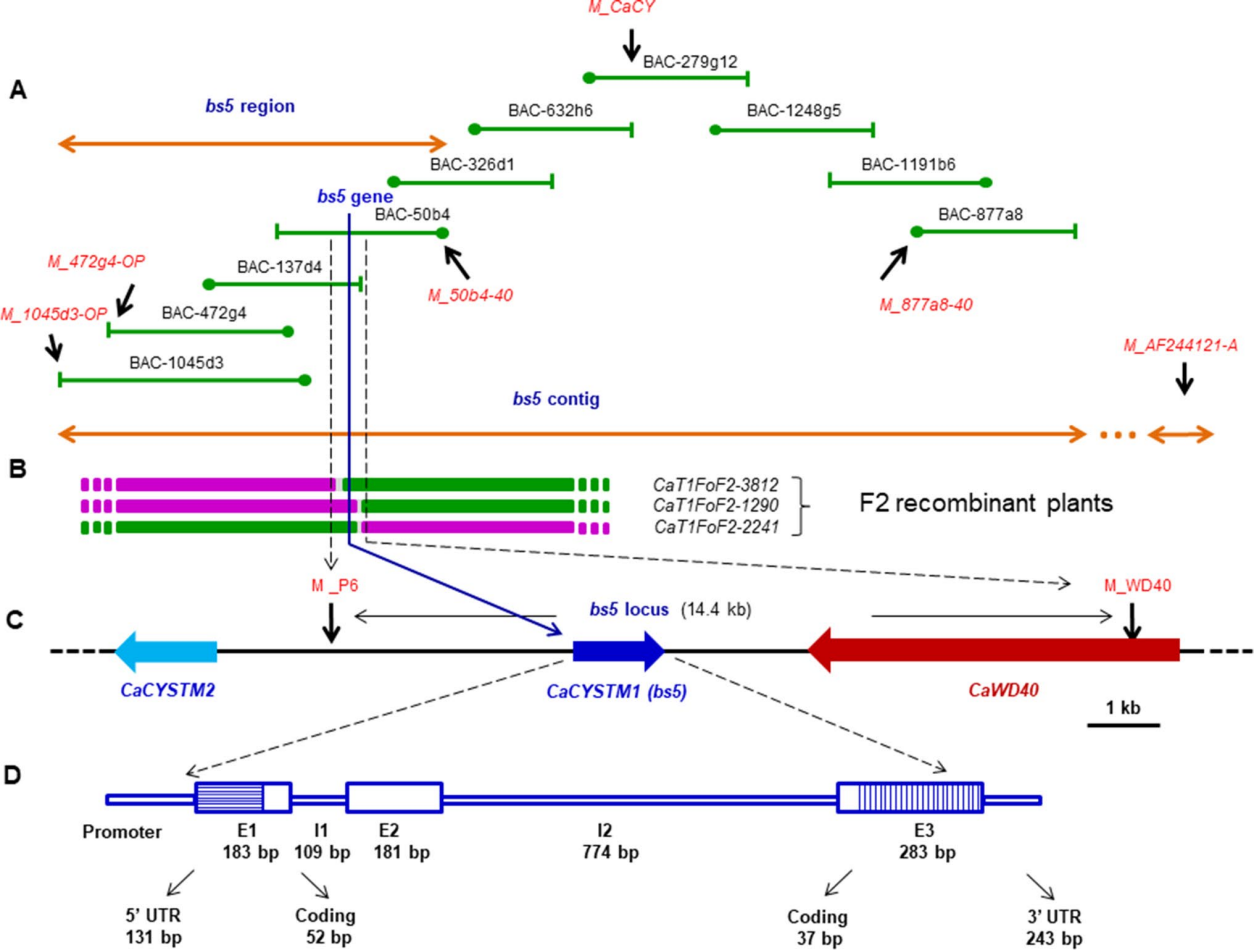
Genetic and physical map around the *bs5 *locus. **A**, Primary and overlapping BAC clones of the bs5 contig are colored green. Positions of markers (red characters in italics), used for positioning the resistant phenotype conditioned by the bs5 gene, are shown black arrows; three overlapping BAC clones (50b4, 137d4 and 472g4) make up the bs5 region. **B**, The chromosome segments of three F2 plant individuals carrying tightly linked recombination breaks around *bs5* are illustrated; colors represent combined genotypes of the two sister-chromatids (Kiss et al. 1998); magenta and green represent resistant and susceptible genotypes, respectively. **C**, gene content within and around the *bs5 *locus flanked by two genetic markers M_P6 and M_WD40; genes and their relative orientations and intergenic regions are shown by horizontal thick arrows and thin black bars, respectively; length of the genes and intergenic regions are to scale. **D**, structure of the *bs5* gene; the length of the exons (E) and introns (I) are shown in base pairs (bp). E1 and E3 are divided by thin vertical bars into 5′ or 3′ untranslated regions (UTR) and coding regions, respectively; length of the exons and introns are in relative scale. OP and -40 ends of the BAC clones are highlighted by vertical bar and closed circles, respectively

Rough genetic mapping was carried out using 8 Xe^S^ and 8 Xe^R^ selected individuals of the F2 segregation population. Total DNA isolated from fresh leaves were subjected to PCR amplification using previously published eleven genetic markers (Lee et al. 2004). The genetic analysis of these eleven pepper specific Simple Sequence Repeat (SSR) markers positioned the *bs5* gene between markers M_CaCY and M_AF244121-A at the upper end of Linkage Group 3 of the genetic map of pepper (Lee et al. 2004). M_CaCY is equivalent to the pepper RFLP marker CaPR10 (Lee et al. 2004) and to the tomato marker M-T1065; (http://www.sgn.cornell.edu/marker/SGN-M1736/details). We extended the mapping population to 4662 F2 individuals to determine the map positions more precisely. The fine mapping revealed that the genetic linkage between marker M_CaCY and *bs5* was about 0.20 ± 0,01 centimorgan. Since marker M_AF244121-A was more distant*,* the marker M_CaCY was a good starting point for chromosome walking toward *bs5* (Fig. 2A).

### Chromosomal walking identifies candidate gene for *Bs5*/*bs5*

The molecular marker M_CaCY was used to isolate the primary BAC clone from the BAC library constructed from pepper CaDH269 plants (see “Materials and methods”). Overlapping BAC clones were isolated using markers generated from the end sequences of the identified BAC clones. These steps were reiterated and resulted in the *bs5* contig of about 600-kilobase covering the *bs5* region (Fig. 2A). Genetic mapping with markers developed based on BAC end sequences delimited the *bs5* gene between markers M_50b4-40 and M_1045d3-OP defining the *bs5* region (Fig. 2A). Based on the sequences of BAC clones BAC-50b4 and BAC-1045d3 (overlapping the *bs5* region), additional genetic markers were developed and used for genetic mapping that further narrowed down the position of the *bs5* gene. This analysis identified three recombinant plants, CaT1Fo-F2-1290, CaT1Fo-F2-2241 and CaT1Fo-F2-3812 (Fig. 2B), which helped to delimit the *bs5* gene in the *bs5* locus flanked by two markers, M_P6 and M_WD40, respectively (Fig. 2C). The sequence of BAC-50b4 between the markers M_P6 and M_WD40 revealed that the *bs5* locus covered 14,654 bp (see partial sequence of BAC-50b4 GenBank accession no.: OM681616). The corresponding *Bs5* locus was also sequenced from the Xe^S^ parent CaFo resulting in 14,734 bp length sequence (GenBank accession no.: OM681615). To identify the genetic alteration responsible for the resistance trait, the sequences of the *Bs5* and *bs5* loci, respectively, were aligned and coding regions were compared (Fig. S5). BLASTN search of the pepper specific Expressed Sequence Tags (EST) database of NCBI (http://www.ncbi.nlm.nih.gov/; taxid:4071) revealed two protein coding sequences, *Ca_WD40* (encoding a protein with WD40 repeats) and *Ca_CYSTM1* (coding for a tail-anchored cystine-rich transmembrane module, CYSTM) genes (Fig. 2C). From the EST sequences of the two genes, TC sequences, *Ca_WD40-TC* and *Ca_CYSTM1-TC*, respectively, were generated (Fig. S6) and aligned to the sequence of *Bs5* and *bs5* locus. Nucleotide polymorphisms were searched for in these two genes to identify mutations distinguishing between the resistant and susceptible alleles. For *Ca_WD40* gene*,* besides several single nucleotide polymorphisms (SNPs) in the non-coding sequence, a single nucleotide change of C to A at position 11 305 was found in the coding sequence between the Xe^S^ and Xe^R^ alleles, but this alteration did not cause a change of amino acid (Fig. S5). In addition, the analysis of the expression of *Ca_WD40* in publicly available RNAseq and EST databases (https://ncbi.nlm.nih.gov/sra/?term=capsicum+annuum+RNA-Seq; https://blast.ncbi.nlm.nih.gov/Blast.cgi) showed similar transcriptional activity, therefore *Ca_WD40* was considered unlikely to be responsible for the Xe^R^ trait. For the *Ca_CYSTM1* gene, several SNPs in the 3′ untranslated region (UTR) and a 6 bp deletion in the third exon were detected between the *Bs5* and *bs5* allele (Fig. S5). Since the 6 bp deletion occurred in the coding sequence of the Xe^R^ allele we suggested that this mutant variant of *Ca_CYSTM1*, denoted *Ca_cystm1* could be a good candidate for the *bs5* gene of pepper.

### Genetic complementation of the *bs5* mutation

**Fig. 3:**
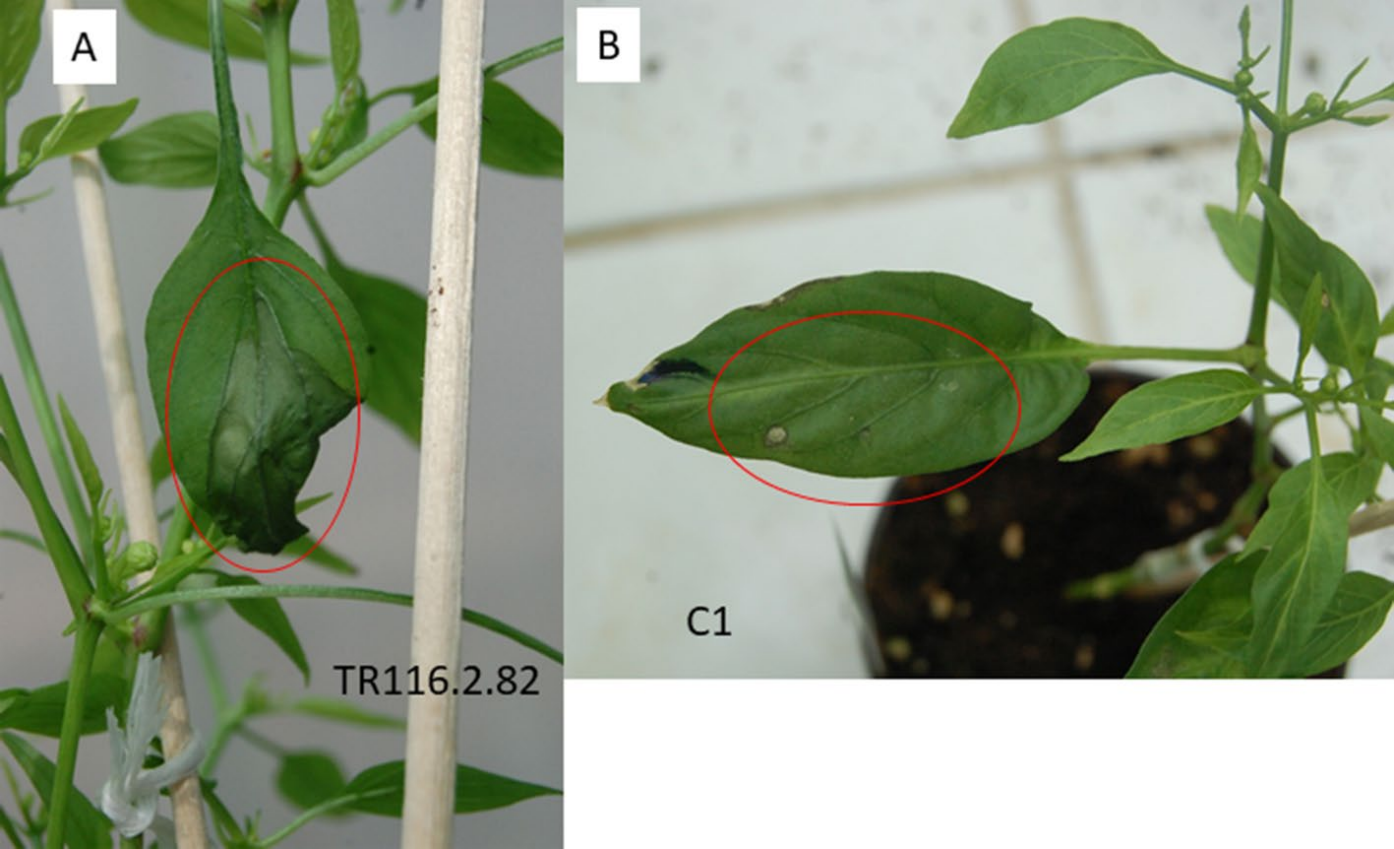
Leaf phenotype of a *Ca_CYSTM1* transformed (**A**) and a control (**B**) plant after inoculation by Xe*78* (inoculation sites are encircled). For TR116.2.82 and C1, see Table S2

**Fig. 4:**
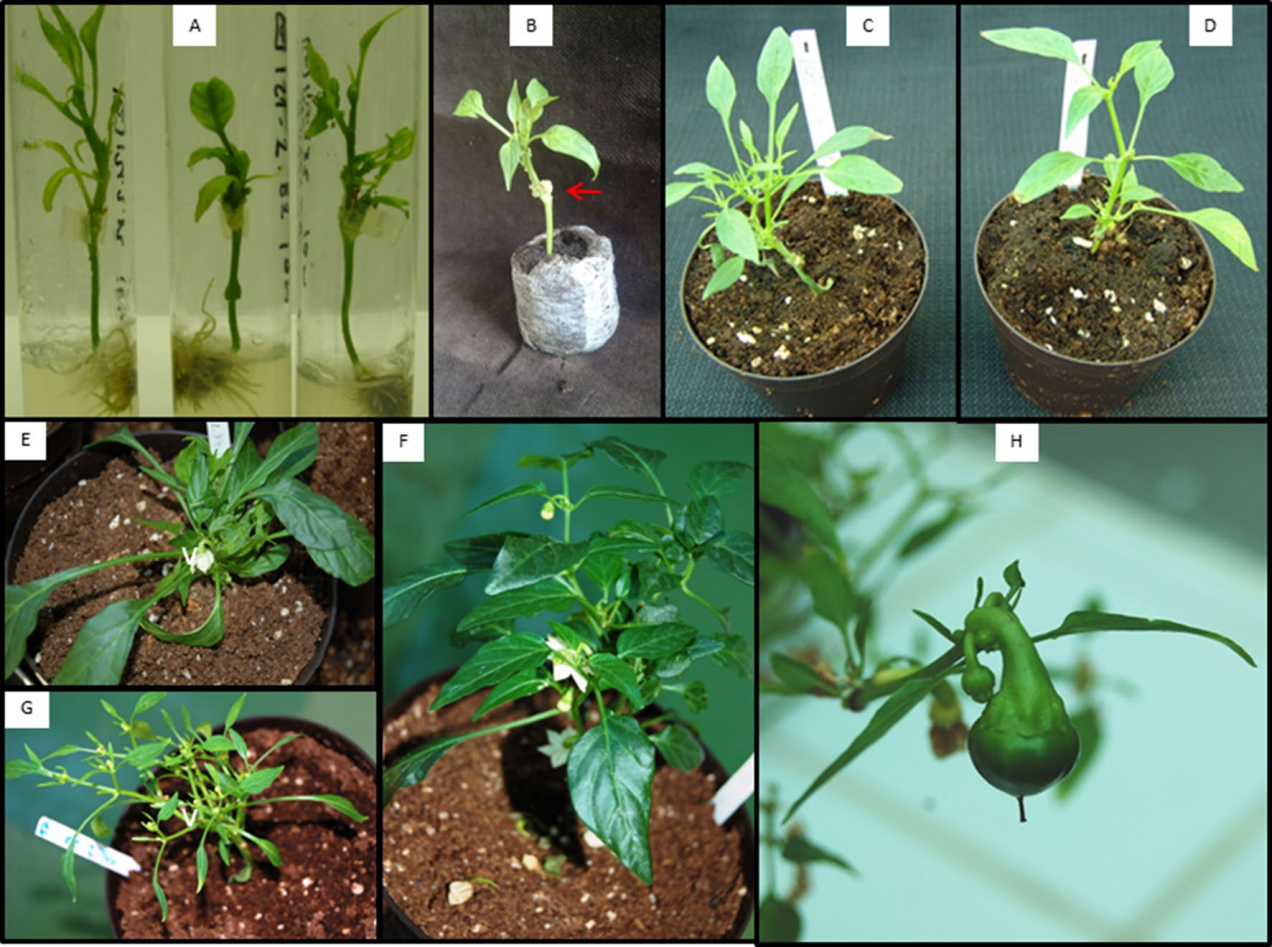
Transformed plants. CaGI plants (which are *bs5*/*bs5* homozygous, *Xe* resistant) were genetically modified by introducing the *Bs5* gene under its native promoter. Five Hyg^R^ (*TR116.2.56*; *TR116.2.82*; *TR116.4.77*; *TR116.4.97*; *TR116.4.103*; *TR116.4.154*) and two Neo^R^ (*TR121.2.19*; *TR121.2.82*) independent transformant shoots were selected on agar matrix after transformation with *pCYSTM1::CYSTM1* constructs. After grafting (**A**), plants were transferred to soil in pots and grown in the greenhouse. Cork-like outgrowth is shown by red arrow. Panels from **C** to **H** show independent transformed plants. **H** shows transformant TR116.2.82, which produced fruits and mature seed

In order to prove that *Ca_CYSTM1* and *Ca_cystm1* corresponded to *Bs5* and *bs5* genes, genetic complementation was carried out. Because the wild type *Bs5* allele—conferring *Xe* susceptibility—is a dominant character, it seemed reasonable to convert the resistant phenotype to sensitivity by introducing the wild type allele into the Xe^R^ pepper line CaGl*.* The sequence of the *Ca_CYSTM1* gene controlled by its native promoter was cloned into pCambia vectors carrying either the hygromycin phosphotransferase resistance (Hyg^R^) or the neomycin phosphotransferase resistance (*Neo*^*R*^) marker gene (Fig. S2) and introduced into the *bs5*/*bs5* homozygous CaGl plants using the ‘shooter’ Agrobacterium transformation system (Mihalka et al. 2003). Leaves developed on transformed plants were tested for the presence of isopentenyl transferase (*ipt*), Hyg^R^, Neo^R^, *Ca_CYSTM1* and *Ca_cystm1* genes*,* respectively, as well as for flower development, mature seed formation and *Xe* susceptibility/resistance (Table S2). Control transformants transformed with empty vector (C1, C2, C3) developed normal roots and could grow to maturity, set seeds and were resistant to *Xe* (Table S2 and Fig. 3B). Positive transformants (*TR116* and *TR121* series) carrying the ectopic Ca_*CYSTM1* gene could only be maintained and developed to full-grown plants when they were grafted to a non-transformed CaGl rootstock. The Xe78-infected leaves on Ca_*CYSTM1* transformants, confirmed for the presence of marker genes as well as Ca_*CYSTM1* and Ca_*cystm1* alleles*,*, respectively, showed the characteristic susceptible symptoms, water-soaked lesions and defoliation (Table S2 and Fig. 3A). We found that transgenic plants carrying the ectopic *CYSTM1* gene were not healthy, in all cases cork-like outgrowth occurred on the stems (Fig. 4A), tissue necrosis occurred on stems and leaves, mortality was high, and in all but one case the plants were sterile; independent transformants are shown in Fig. 4B–H.

From the map-based cloning results and the genetic complementation experiments, we concluded that the 6 bp deletion found in the *Ca*_*CYSTM1* gene was in fact responsible for the Xe^R^ trait in pepper, consequently Ca_*CYSTM1* and Ca_cystm1 gene corresponded to *Bs5* and *bs5* genes, respectively. Therefore, the term *Bs5* and *bs5* will be used hereafter*.*

### Expression of the *CYSTM* genes in pepper

Expression of *Bs5* and *bs5* genes was investigated by whole transcriptome shotgun sequencing (RNA Seq) of RNA isolated from leaf tissue of 8 Xe^R^ and 8 Xe^S^ bulk plants of the CaFoT1-F2 population (the same ones used for preliminary mapping). This analysis revealed that both *Bs5* and *bs5* genes were expressed in leaf tissue (data not shown). In addition, *Bs5* is expressed in other tissues as is supported by EST sequences (Table S3) and RNA Seq data of Sequence Read Archives of NCBI (e.g., SRX15644350-SRX15644367, SRX13447132-SRX13447138, SRX13447140-SRX13447147, SRX9188483-SRX9188486). It is worth mentioning that there is no nucleotide difference between the genomic, EST and RNASeq sequences concerning *Bs5* gene despite the sequences originated from different pepper cultivars e.g., (ECW50R, CaT1, CaFo, Ca cv. Bukang, Ca cv. Nokkwang and Ca cv. Hang Keun, respectively).

### The pepper genome contains* a CYSTM* paralog of *Bs5*

A similarity search using *Bs5*-*TC* as query identified an additional homologous gene, *Ca_CYSTM2* encoding another CYSTM protein, Ca_CYSTM2 (Fig. S6, Fig. S7, Fig. S8). The *Ca_CYSTM2* gene is located on the other side of the *bs5* gene compared with *Ca_WD40* positioned in opposite orientation indicating the duplication of the *CYSTM* genes in pepper. ESTs for *Ca_CYSTM2* gene were obtained from the pepper EST database (taxid:4072) of NCBI (Table S3) and the alignment of *Bs5*-*TC* and *Ca_CYSTM2-TC* sequences at nucleotide level (Fig. S7) revealed extensive similarity throughout the coding region (256 nucleotides out of 273 were identical; 93.8% homology). It is interesting to note, that, out of the 17 nucleotide changes, seven were non-synonymous and ten were synonymous mutations (Fig. S7) consistent with *Ca_CYSTM2* being under strong purifying selection (Lawrie et al. 2013). However, the ~ 300 bp promoter region of *Bs5* and *Ca_CYSTM2* displayed considerable differences that might indicate distinct transcriptional activity of the two *Ca_CYSTM* genes.

### The bs5 protein is deficient in the predicted transmembrane domain

**Fig. 5:**
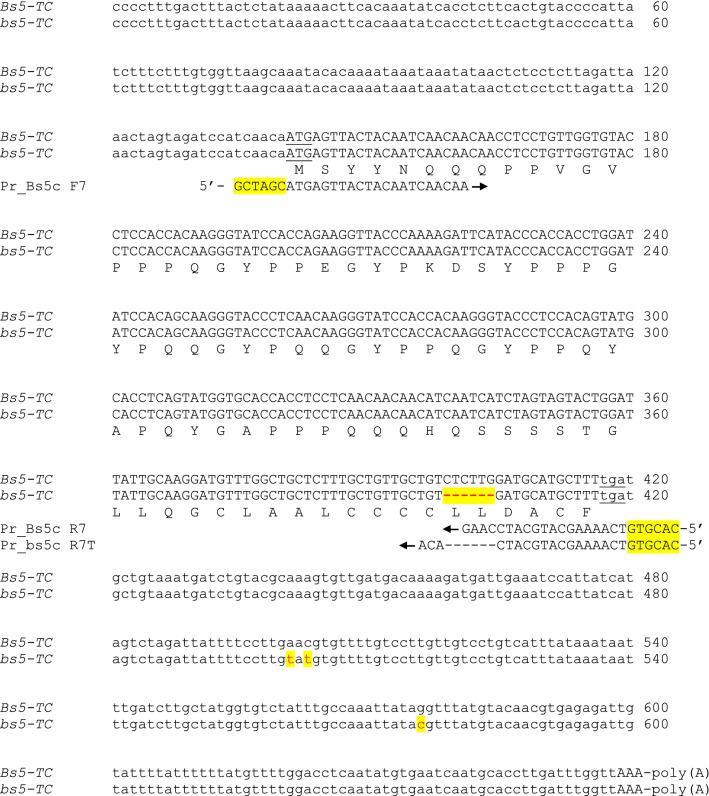
Alignment of cDNA and deduced protein sequence of *Bs5* and *bs5* alleles*.* The sequences of *Bs5-mRNA* and *bs5-mRNA* were taken from Fig. S6, and represent coding, 5′- and 3′-UTR sequences of *Bs5* and *bs5* alleles, respectively. The sequences of *Bs5-mRNA* and *bs5-mRNA* were verified also by sequencing both parental genomic DNAs and coding sequences (see text). Except for the six base pair deletion, *bs5* has the same amino acid sequence as *Bs5*. Start and Stop codons are underlined. Deletion is highlighted by red dashes (**-**). poly(A) represents the polyA tail. Unmatched nucleotides are highlighted with red characters. The primers aligned to the sequence were used to synthetize cDNA. The direction of primers is indicated by black arrowheads and by 5′ marking

**Fig. 6:**
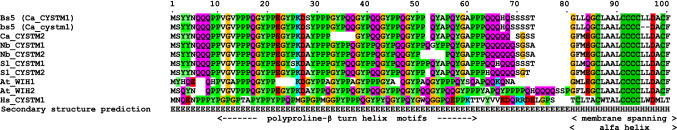
Multiple alignment of CYSTM proteins from selected organisms, and secondary structure prediction. Protein sequences were obtained from the protein databanks as described below. Alignment was done manually by the help of aligned coding sequences. TM domains were predicted by the “Split Server Prediction” software: http://split.pmfst.hr/split/4/. The 2 amino acid deletion in bs5 (second row) is highlighted be two dashes. The amino acids were highlighted by colors according to the scheme by Lesk (http://www.bioinformatics.nl/~berndb/aacolour.html) as follows: small nonpolar (orange), G; hydrophobic (green), C, P, Y; polar (magenta), Q; negatively charged (red), D, E; positively charged (blue), K,R. The names of the sequences are labeled as follows: the first four letters denote the abbreviated name of the organism (first letter stands for the first letter of the genus name, the next three are the first three letters of the species name). After this the name of the CYSTM proteins were given. The species abbreviations are: Ca: *Capsicum annuum*; Nb: *Nicotiana benthamiana*; Sl: *Solanum lycorpersicum*; At: *Arabidopsis thaliana*; Hs: *Homo sapiens.* The protein sequences were obtained for: Bs5, bs5, Ca_CYSTM2, this study; Slyc_CYSTM1 and Slyc_CYSTM2: solgenomics.net reference no.: Solyc09g098310.3.1 and Solyc09g098300.3.1, respectively; Nb_CYSTM1 and Nb_CYSTM2, see Fig S9, respectively; At_WIH1 and At_WIH2: NCBI Reference Sequence: NP_569052.1 and NP_181673.1, respectively; Hs CYSTM1: https://www.uniprot.org/uniprot/Q9H1C7. Abbreviation of the secondary structure predictions (https://predictprotein.org/): E, extended or other; H, Alpha-helix

*Bs5* and *bs5* genes code for peptides of 92 and 90 residues, respectively (Fig. 5). Similarity search of the predicted peptide sequence indicated that both are tail-anchored cystine-rich transmembrane module (CYSTM) proteins (Venancio et al. 2010). A transmembrane (TM) domain was predicted at the C-terminus of the Bs5 protein (Fig. 6) composed of 20 AA residues indicating membrane localization. The mutant *bs5* encodes a shorter version of Bs5 lacking two residues in the predicted TM domain (Fig. 5 and Fig. 6) which probably affects the function of the bs5 peptide. Similarity search at the protein level identified homologous genes in other *Solanaceae* (Fig.S9) and more distant plant species but even a homologous peptide was found among human translated sequences (Fig. 6).

### The Bs5-GFP fusion protein was localized to the plasma membrane

**Fig. 7:**
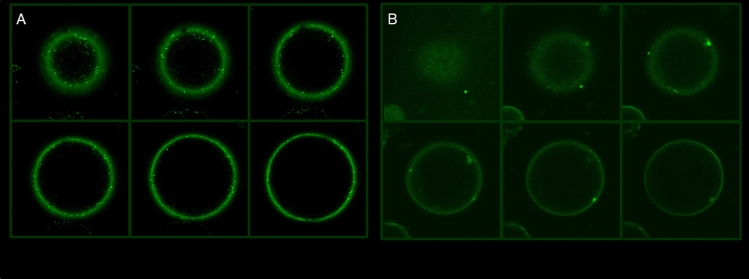
Confocal microscopy of N-terminal GFP-Bs5 (**A**) and GFP-bs5 (**B**) fusion proteins. For GFP fusion constructs, *Bs5* and *bs5* coding sequences were amplified from cDNA using specific primers, Pr_Bs5c F7, Pr_Bs5c R7 and Pr_Bs5c F, Pr_bs5c R7T, respectively, containing *NheI* and *PmlI* restriction endonuclease sites (Fig. S3) and cloned at the C-terminal end of the green fluorescent protein (GFP) of pCambia-1302. The resulting plasmids pCambia-1302*-*GFP*-Bs5* and pCambia-2302-GFP-*bs5* (see legend to Fig S3) were infiltrated into young *Nicotiana benthamiana* leaves. After 48 h, protoplasts were prepared from the infected area using the protoplast isolation method described by Nagy and Maliga (1976) and the subcellular localization of GFP fluorescence was investigated by Zeiss (Zeiss LSM 510 META) and Olympus (Olympus Fluoview FV1000) Confocal laser scanning microscopes

Tail-Anchored proteins can have a variety of membrane locations (Abell et al. 2003; Venancio and Aravind 2010; Abell and Mullen 2011). To define what membrane the Bs5/bs5 proteins are located in subcellularly, constructs coding for green fluorescent protein (GFP) fused to the N-termini of *Bs5* or *bs5* peptides were generated (Fig. S3). The constructs were transiently expressed in *Nicotiana benthamiana* leaves, protoplasts were isolated from the infected tissues and the signal of the GFP fusion proteins of Bs5/bs5 was visualized by confocal laser scanning microscope. High-resolution optical images captured from different depths of the sample demonstrated that N-terminal GFP-Bs5 and GFP-bs5 fusion proteins are targeted into the plasma membrane by slightly different efficiency (Fig. 7, Panel **A** and Panel **B**).

## Discussion

In this paper, we described the map-based cloning of the *bs5* gene of pepper which conditions recessive resistance against *Xanthomonas euvesicatoria*. In pepper plants carrying the *bs5* allele in homozygous configuration, the leaf tissue at the site of infection, remains alive and functioning. This phenotype is substantially different from that of the programmed cell death-based hypersensitive response (HR) characterized by tissue necrosis (Klement et al. 1964; Balint-Kurti 2019). In addition, *bs5* based resistance is stable; no breakdown has yet been observed. This is an important feature of recessive alleles of susceptibility genes which confer resistance.

Reverse genetic complementation was taken to substantiate *bs5* as responsible for resistance. In these complementation experiments *bs5* homozygous CaGl plants were transformed with wild the type *Bs5* allele. Transformants with ectopic *Bs5* displayed abnormal phenotypes in contrast to the control transformants (without the *Bs5* gene), which were normal in appearance. The reason for the altered habitus of the *Bs5* transformants is not known. One could hypothesize that attenuated function of the Bs5/bs5 proteins might be responsible for the abnormal phenotype as a consequence of gene silencing. RNA interference provoked by the ectopic *Bs5* transgene could plausibly lower the expression of the *Bs5* and *bs5* genes. This same mechanism could also reduce the amount of the *CaCYSTM2* gene product, since there is extensive nucleotide sequence similarity between that of *Bs5* and *CaCYSTM2* genes (Fig S7). The simultaneous silencing of *Bs5*, *bs5* and *CaCYSTM2,* might be responsible for insufficient function(s) of the corresponding proteins leading to morphological changes. *wih1*, *wih2* and *uvi15* all encode mutant CYSTM proteins homologous to Bs5 (Fig. 5; Venancio and Aravind 2010) and a similar stunted character with a high percentage of sterility and abnormal growth was demonstrated for *Arabidopsis thaliana* plants carrying *wih1/wih2* double mutations (Lieber et al. 2011). Likewise, sensitivity to ultraviolet light, defective sporulation, and loss of viability during growth of the *Schizosaccharomyces pombe uvi15* mutant has also been described (Lee et al. 1994, 1995). Taking into account the abnormal mutant phenotypes one could conclude, that CYSTM proteins have a more general function than stress tolerance (Lee et al. 1995; Beilharz et al. 2003; Li et al. 2009; Venancio and Aravind 2010; Hillenmeyer et al. 2010; Feng et al. 2011). The available CYSTM null mutants in *Arabidopsis wih1/wih2* double mutants (Lieber et al. 2011) and in *Schizosaccharomyces pombe uvi15* mutant, as well as the *Bs5* transformed pepper lines (see above), were all viable but of decreased fitness indicating that CYSTM proteins are required for normal growth.

DNA sequence analysis of the wild type *Bs5* gene revealed that *Bs5* has three exons and codes for a short protein of 92 amino acids (Fig. S5, Fig. 6). The Bs5 protein belongs to the tail-anchored (TA), cysteine-rich transmembrane module (CYSTM) protein family (Kutay et al. 1993; Lee et al. 1995; Beilharz et al. 2003; Li et al. 2009; Venancio and Aravind 2010; Abell and Mullen 2011; Feng et al. 2011; Lieber et al. 2011). The *bs5* allele has the same exon–intron structure and encodes a similar protein to Bs5 except for the lack of two leucine amino acids in the TM domain (Fig. 5, 6). This shorter protein seems to be responsible for the resistance trait in *bs5* pepper lines.

Tail-Anchored proteins are targeted to membranes in such a way that the N-terminal ends face the cytosol (Paul et al. 2013) and the C-terminal part is inserted into the membrane. Taking into consideration this feature, N-terminal GFP-Bs5 protein fusions were generated and used to transiently express these constructs in *N. benthamiana* (*Nb*) leaves. Although *Nb* was a heterologous system, but is in the same family as pepper, we expected that Bs5/bs5 proteins should be targeted into the same membrane in *Nb* as in *Ca*, since AAs in the N-terminal and the TM domain direct TA proteins into the membrane system (Borgese et al. 2003). From the alignment of the CYSTM proteins, one can see that the first 24 AAs and the last 16 AAs of Bs5 and NbCYSTM, respectively are identical (Fig. 6). According to the microscopic images, both GFP-Bs5 and GFP-bs5 protein were localized in the cell membrane, which is in agreement with previous publications for the location of CYSTM proteins (Borgese et al. 2003; Abell et al. 2007; Kriechbaumer et al. 2009). On the other hand, GFP-Bs5 and GFB-bs5 proteins, respectively, did not show exactly the same intensity and localization pattern of GFP, since GFP-Bs5 protein localized more or less entirely in the cell membrane while in the case of GFP-bs5 membrane targeting was disturbed to some extent and GFP signals displayed a fainter and more diffuse signal (Fig. 7). Confocal microscopy of the GFP-Bs5-transformed leaves also revealed several dark green spots near and around the membrane, suggestive of some kind of compartmentalization and vesicular trafficking of the GFP-Bs5 protein (Fig. 7A). These dark green spots were almost missing in the case of the GFP-bs5 fusion (Fig. 7B). The lack of the two adjacent leucines in bs5 therefore disturbed the normal membrane targeting process and abolished the dark green spots characteristic of GFP-Bs5. The above findings were in agreement with results described in previous publications showing that CYSTM TA proteins were cell membrane located in a wide range of organisms (Xu et al. 2018; https://www.uniprot.org/uniprot/Q9H1C7).

It is interesting to note, that in spite of high degree of protein sequence similarity (or identity) (Fig. 6) and similar expression in leaves of both *CaCYSTM2* and *bs5*, *CaCYSTM2* did not complement *bs5* with respect to resistance in *bs5* plants (ECW50R, CaT1, CaDH269 and CaGl). Non-complementation might be attributed to the three AAs difference, LLQ in *bs5* and FME in *CaCYSTM2*, respectively at the beginning of the TM domain (Fig. 6). From this, one may conclude that *CaCYSTM2* is not a functional paralogue of *Bs5*/*bs5.*

The molecular function of the CYSTM proteins is unknown. One can only speculate that the TA proteins should be involved in those biological processes—such as biotic or abiotic stresses, plant development (Lee et al. 1995; Venancio and Aravind 2010; Xu et al. 2018; Lieber et al. 2011)—which one can measure, visually see and/or deduce from the phenotype and properties of the mutant organisms (e.g., phenotypes of *A. thaliana wih1*/*wih2*, *S. pombe uvi15* and *Ca bs5* mutants, respectively). AA sequence resemblance, interaction with other proteins as well as the localization of the CYSTM proteins, however, may leave some more room for cautious speculation. The cytoplasmic domain of Bs5 contains glycine-tyrosine-proline-proline repeats (GYPP-R). These repeats are found in several proteins and represent one class of proline rich proteins which can participate in protein–protein interactions with high specificity (Matsushima et al. 1990; Williamson 1994; Brownawell and Creutz 1997; Kay et al. 2000; Strugnell et al. 2005; Hu et al. 2009; Creutz 2009; Li et al. 2009; Feng et al. 2011). The C-terminal TM domain indicates, and experimental evidence verifies, that CYSTM TA proteins are membrane bound (see also Fig. 7). It is plausible to suppose that CYSTM proteins may interact with other proteins. If identified, interacting partners might shed light on the biological process in which the complex participates (Huang et al. 2013). For example, the human CYSTM1 protein (https://www.uniprot.org/uniprot/Q9H1C7) (and see also Fig. 5) seems to interact with two proteins, BAG3 (https://www.uniprot.org/uniprot/O95817) (a molecular chaperone regulator) and SYT16 (https://www.uniprot.org/uniprot/Q17RD7) (Synaptotagmin-16), which were identified in yeast two-hybrid system. BAG3 may be involved in chaperone binding, while SYT16 participates in vesicular trafficking and exocytosis of secretory vesicles in non-neuronal tissues of mammals (Wolfes and Dean 2020). Besides, the TM domain of the CYSTM proteins adopts an α-helix conformation in the membrane, and as a consequence cysteine residues face in different orientations allowing the establishment of disulfide or hydrogen bonds with residues of neighboring proteins, as was demonstrated for Syntaxin A1, a CYSTM TA protein (https://www.uniprot.org/uniprot/Q16623; Gregoret, et al. 1991; Cohen et al. 2007; Witt 2008; Bachnoff et al. 2013). Syntaxins and annexins function in vesicle fusion process (Brownawell and Creutz 1997; Vardar et al. 2016). As a conclusion, it cannot be excluded, that the Bs5 protein, one way or another, participates in vesicular trafficking (e.g., endocytosis).

It has been demonstrated that during *Xe* infection effector molecules from *Xe* enter plant cells and are delivered through translocons which are the pore forming apparatus of the Type Three Secretion System (TTSS) (Kim and Hartmann 1985; Schornack et al. 2008; Kay and Bonas 2009; Büttner and Bonas 2010). Translocons, are formed in the plant cell membrane and built up by proteins encoded by the pathogen (Sory and Cornelis 1994; Jones and Dangl 2006; Block et al. 2008; Büttner and He 2009; Galan et al. 2014). This arrangement necessitates the interaction of proteins from both partners. If a plant protein involved in this interaction is altered by a mutation in such a way that the effector delivery is disturbed, then the plant may become resistant to *Xe*. It is possible, that the wild type Bs5 protein is involved in the transport of effectors—in one way or another—into the plant cells and its mutant bs5 allele will be non-functional in effector delivery. Hampered effector entry was shown in *bs5* plants compared with the susceptible pepper lines (Ortega et al. 2019), therefore we hypothesize that *bs5* plants are resistant against *Xe* due to the lack of entry of *Xe* effectors into the host cell. Nevertheless, it is plausible to suppose that resistance to *Xe* may be generated by inducing deletions in orthologous *CYSTM* genes using genome editing approach (Osakabe and Osakabe 2015). The same 6 bp deletions could result in *Xe* resistant plants in those plant species, e.g., in tomato (*Solanum lycopersicum*), which are closely related to pepper and in which no durable natural resistance exists against *Xanthomonas euvesicatoria*.

## Supplementary Information

Below is the link to the electronic supplementary material.

Supplementary file 1: **Fig. S1** Genotypes of the plants used in allelism tests Marker M_bs5g were used to genotype the plants for *Bs5*/*bs5* alleles. Total DNA isolated from leaves of the plants was used as templates. After PCR amplification using Pr_bs5g F1 and Pr_bs5g R1, fragments were separated in agarose gel, and visualized bands were converted to genotypes. Upper and lower fragments were amplified from *Bs5* and *bs5* alleles, respectively. In heterozygotes where the two alleles were amplified the heteroduplex fragments run slower, therefore the upper band is fuzzy. The resistant and susceptible phenotypes of the self-pollinated progenies of the above plants verified the heterozygous and homozygous genetic configuration (data not shown). The genotypes of the plants for *Bs5*/*bS5* alleles using M_bs5g marker are as follows: ECW50R, *bs5/bs5;* T1xECW50R F1, *bs5/bs5;* CaT1, *bs5*/*bs5;* T1xFo F1, *Bs5/bs5;* CaFo, *Bs5/Bs5;* CaDH269 (*bs5*/*bs5*); CaGl (*bs5*/*bs5*), ECW, (*Bs5*/*Bs5*).

Supplementary file 2: **Fig. S2.** Structural and functional map of *Bs5* genomic constructs for *Agrobacterium* mediated transformation. Abreviations: *Hyg*^*R*^, hygromycin phosphotransferase gene; *Neo*^*R*^, neomycin phosphotransferase gene; UTR, Untranslated region. *GUS*, β-glucuronidase gene, *GFP*, Green Fluorescence Protein gene, *Nos*, Nopaline synthase terminator, *35S*, *CAMV 35S* gene, term, transcription terminator. Arrows indicate the direction of transcription, RB and LB, Right and Left border sequence, respectively

Supplementary file 3: **Fig. S3** N-terminal GFP-Bs5, GFP-bs5 constructs used for confocal microscopy. The double stranded cDNA sequence of *Bs5* and *bs5* was amplified from single stranded cDNA synthetized from polyA^+^ RNA isolated from young leaves of CaFo and CaT1 parental plants, respectively. For amplification, primer pairs Pr_Bs5c F7, Pr_Bs5c R7, and Pr_Bs5c F7, Pr_bs5 R7T, respectively, were used. Isolation of polyA + RNA and cDNA synthesis were carried out with Promega SV total RNA Kit and RevertAid First Strand cDNA Synthesis Kit. Double stranded cDNA sequences of CaFo*-Bs5* and CaT1*-bs5* were cloned into pGem-T Easy vector. From these constructs, CaFo *Bs5* and CaT1 *bs5* cDNA sequences were cloned in frame after the structural gene of green fluorescent protein (GFP) of pCambia-2302 vector using NheI, SalI and NheI, BstEII restriction enzymes (SalI and BstEII sticky ends were made blunt ended by Mung Bean nuclease. Panel **A** and **B** shows the functional and relevant restriction map of pCambia-2302 CaFo *Bs5* and pCambia-2302 CaT1 *bs5*, respectively. The nucleotide and amino acid (AA) sequences of the junctions at the *GFP* – *Bs5*, and *GFP*—*bs5* genes/proteins are shown in Panel **C** and **D**, respectively. Primer sequences are underlined.

Supplementary file 4: **Fig. S4** Demonstration of the hybrid nature of the F1 plants from cross CaT1 (**♀**) x CaFo (♂) using molecular marker M_472g4-OP. Fragment length polymorphism was detected after PCR amplification and agarose gel electrophoresis. Total DNA isolated from leaves of the F1 plants were amplified with primers Pr_472g4-OP F and Pr_472g4-OP R. Amplification products were separated in 3% agarose gel, and visualized bands were converted to genotypes. Upper and lower fragments were amplified from *Bs5* and *bs5* alleles, respectively. MM, 100 bp molecular ladder (Fermentas). F1 hybrid plants are numbered from 1- 46. CaT1*,* Ca var. T1; CaFo*,* Ca *cv. *Feherozon

Supplementary file 5: **Fig. S5** Alignment of the nucleotide sequence of the *Bs5* and *bs5* loci. The sequence of the *bs5* locus was determined by sequencing *Eco*RI and *Hind*III subclones as well as by high throughput SOLiD™ sequencing of BAC-50b4. The wild type *Bs5* locus was sequenced from amplified genomic DNA of plant *CaFo* using specific primer pairs. These primer pairs were designed in such a way that the amplified fragments were overlapped with each other. The sequences of the *bs5* and the *Bs5* loci were deposited to the GenBank (GenBank accession no.: OM681616; GenBank accession no.: OM681615, respectively). The sequence of *Bs5-TC*, *CaWD40-TC* and primers for *markers M_P6, M_WD40, M_bs5g, M_Bs5g were* also aligned to the genomic sequence. *CaBs5-TC* and *CaWD40-TC* sequences were presented by red characters. Start and Stop codons were underlined. 5′-UTR/3′-UTR and coding regions were highlighted by lower and upper case letters, respectively. Introns and missing nucleotides (deletions) are indicated by dashes. The direction of primers is indicated by black arrows and by marking the 5′ end. Nucleotide alterations and indels in the sequence of *bs5* gene were highlighted by turquoise-blue background color. poly(A), Polyadenylation tail

Supplementary file 6: **Fig. S6** Tentative consensus sequence of the *CYSTM* and WD40 genes in pepper. The > 14 kb partial genomic sequence of BAC-50b4 between the flanking genetic markers (M_P6, M_WD40) was used to search homologous sequences in the NCBI “Expressed Sequence Tags” (EST) databases using *BLASTN* option (http://www.ncbi.nlm.nih.gov/). EST sequences of the five genes were recruited from the *Capsicum annuum* (taxid:4072) EST database of NCBI (http://www.ncbi.nlm.nih.gov/) using the *Bs5* region of BAC-50b4 (see Fig. 1). The individual ESTs (see Table S3) were aligned (using the SeqMan program of the DNA STAR Software; http://www.dnastar.com/), and the consensus sequence generated by the program was taken as the Tentative Consensus (TC; Quackenbush et al. 2000) sequences for *Bs5*, *Bs5-TC* (Panel **A**), for *WD40, CaWD40-TC* (Panel **B**) and for *Ca_CYSTM2-TC* (Panel **C**). No nucleotide difference was found between the genomic, *CYSTM1*-*TC* and *CYSTM1-mRNA* sequences despite the genomic and EST sequences originated from different pepper cultivars (CaFo, Ca cv. Bukang, Ca cv. Nokkwang and Ca cv. Hang Keun, respectively). Aligning *CYSTM1-mRNA* and *cystm1-mRNA* revealed the 6 bp difference between the two sequences

Supplementary file 7: **Fig. S7** Alignment of *Ca_CYSTM2-TC and Bs5-TC*. The sequences were taken from Fig. S3, and represents coding, 5′- and 3′-UTR sequences, respectively. Single letter coded amino acids (AA) were deduced from Ca_CYSTM2-TC sequence*.* Start and Stop codons were underlined. 5′-UTR/3′-UTR and coding regions were highlighted by upper and lower case letters, respectively. Unmatched nucleotides are highlighted with red characters. Nucleotide changes in the coding region which were in the third position of the codon and resulted in no AA change were underlined. The first and second AA in a pair marked deduced AAs from the Ca_CYSTM2-TC and Bs5-TC sequence, respectively. Amino acid similarity highlighted in the paired AA by color: hydrophobic, green; small nonpolar, orange; polar, magenta

Supplementary file 8: **Fig. S8** Alignment of the promoter and 5′ UTR region of *Bs5/bs5* and *Ca_CYSTM2* genes. More than 300 bp sequence upstream of the start codon was aligned by the help of dot plot matrix based homology search. Sequences before the start codon are in lower case letters. The length of the 5′ UTR sequences were defined according to the longest cDNA at the 5′ end. 5′ UTR sequences are in italics. Identical nucleotides are shown by black characters, mismatch nucleotides are in red. *Bs5* and *bs5* sequences were 100% identical in this region and displayed as *Bs5/bs5*. “pr” after the name of the genes stands for promoter

Supplementary file 9: **Fig. S9.** The coding and protein sequence of the CYSTM genes of *Nicotiana benthamiana*. The coding sequences of *Nb_CYSTM1* and *Nb_CYSTM2* were retrieved from the SolGene database (https://solgenomics.net/) contigs Niben101Scf02563Ctg059 and Niben101Scf02915Ctg016/Niben101Scf02915Ctg017, respectively. The coding sequence was translated to obtain the deduced protein sequence

Supplementary file10 (DOCX 15 kb)

Supplementary file11 (DOCX 77 kb)

Supplementary file12 (DOCX 15 kb)

## Data Availability

All data relevant to this study have been provided in the manuscript, or in the supplemental online materials. Sequence data of the Bs5 and bs5 loci described in this article can be found in the GenBank/EMBL sequence collection under the accession numbers OM681616 and OM681615, respectively. All plant materials in this study are available for research purposes from the corresponding author by request.
