# Correction to: The *bs5* allele of the susceptibility gene *Bs5* of pepper (*Capsicum annuum* L.) encoding a natural deletion variant of a CYSTM protein conditions resistance to bacterial spot disease caused by *Xanthomonas* species

**DOI:** 10.1007/s00122-023-04366-2

**Published:** 2023-05-08

**Authors:** Zoltán Szabó, Márta Balogh, Ágota Domonkos, Márta Csányi, Péter Kaló, György B. Kiss

**Affiliations:** 1 Institute of Genetics and Biotechnology, Hungarian University of Agriculture and Life Sciences, Szent-Györgyi A. U. 4., 2100 Gödöllő, Hungary; 2 AMBIS Biotechnology Research and Development Ltd., Budapest, Hungary; 3 Institute of Plant Biology, Biological Research Center, Eötvös Lóránd Research Network, Szeged, Hungary

**Correction to: Theoretical and Applied Genetics (2023) 136:64** 10.1007/s00122-023-04340-y

In the second but last paragraph of the Discussion, Fig. 5 was wrongly referred to. The correct text and reference read:

“For example, the human CYSTM1 protein (https://www.uniprot.org/uniprot/Q9H1C7) (and see also Fig. 6) seems to interact with two proteins, BAG3 (https://www.uniprot.org/uniprot/O95817) (a molecular chaperone regulator) and SYT16 (https://www.uniprot.org/uniprot/Q17RD7) (Synaptotagmin-16), which were identified in yeast two-hybrid system. BAG3 may be involved in chaperone binding, while SYT16 participates in vesicular trafficking and exocytosis of secretory vesicles in non-neuronal tissues of mammals (Wolfes and Dean 2020).”

In the original version of article, the authors’ Conflict of Interest statement was incomplete. The correct statement should read:

**Conflict of interest**: Ágota Domonkos, Márta Csányi, and Péter Kaló declare no conflicts of interest. Zoltán Szabó, Márta Balogh and György B. Kiss declare that they are inventors in the patent WO2014068346A3 „Identification of a *Xanthomonas euvesicatoria* resistance gene from pepper (*Capsicum annuum*) and method for generating plants with resistance.

