# Supplementary figures and images for: The bs5 allele of the susceptibility gene Bs5 of pepper (Capsicum annuum L.) encoding a natural deletion variant of a CYSTM protein conditions resistance to bacterial spot disease caused by Xanthomonas species
Source: Theor Appl Genet. 2023 Mar 21;136(3):64. doi: 10.1007/s00122-023-04340-y (PMC10030403; doi:10.1007/s00122-023-04340-y)

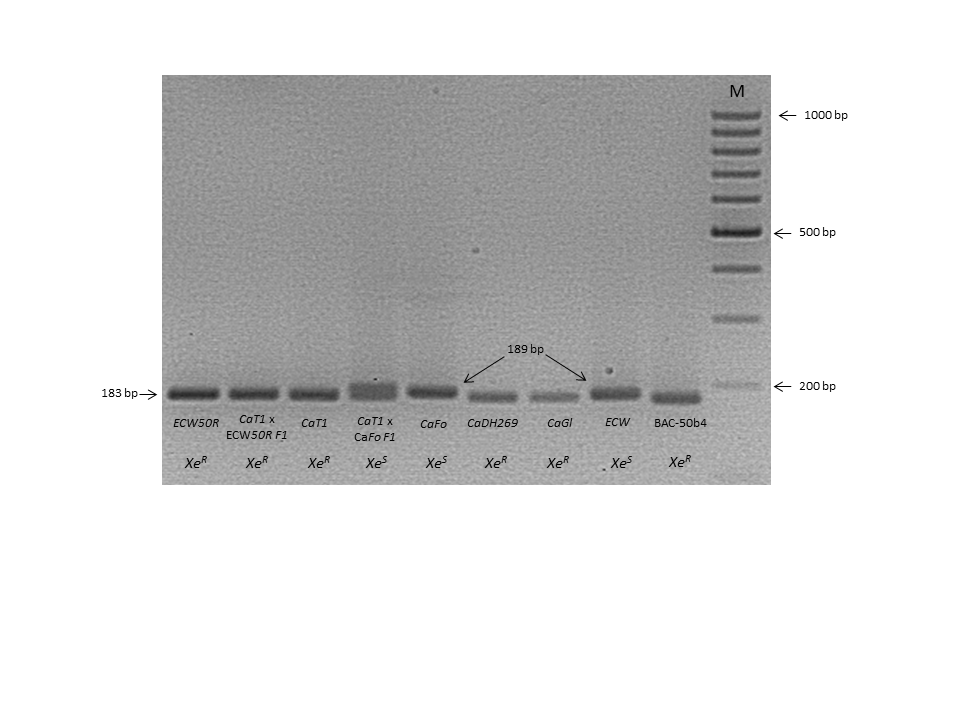

Supplement: Supplementary file 1 — Fig. S1 Genotypes of the plants used in allelism tests Marker M_bs5g were used to genotype the plants for Bs5/bs5 alleles. Total DNA isolated from leaves of the plants was used as templates. After PCR amplification using Pr_bs5g F1 and Pr_bs5g R1, fragments were separated in agarose gel, and visualized bands were converted to genotypes. Upper and lower fragments were amplified from Bs5 and bs5 alleles, respectively. In heterozygotes where the two alleles were amplified the heteroduplex fragments run slower, therefore the upper band is fuzzy. The resistant and susceptible phenotypes of the self-pollinated progenies of the above plants verified the heterozygous and homozygous genetic configuration (data not shown). The genotypes of the plants for Bs5/bS5 alleles using M_bs5g marker are as follows: ECW50R, bs5/bs5; T1xECW50R F1, bs5/bs5; CaT1, bs5/bs5; T1xFo F1, Bs5/bs5; CaFo, Bs5/Bs5; CaDH269 (bs5/bs5); CaGl (bs5/bs5), ECW, (Bs5/Bs5). [file 122_2023_4340_MOESM1_ESM.tif]

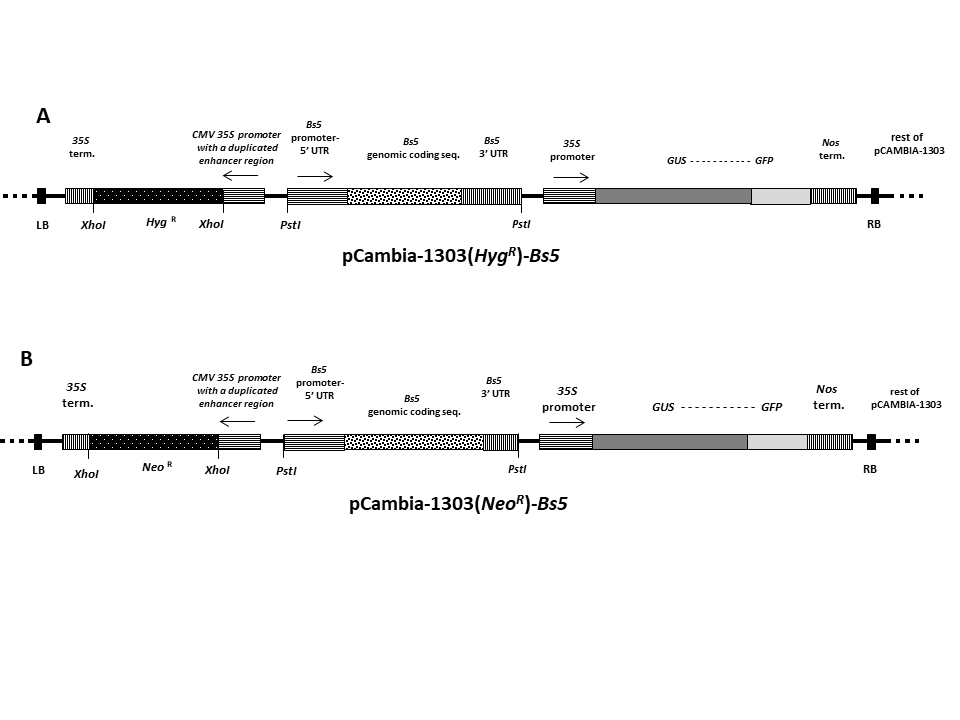

Supplement: Supplementary file 2 — Fig. S2. Structural and functional map of Bs5 genomic constructs for Agrobacterium mediated transformation. Abreviations: HygR, hygromycin phosphotransferase gene; NeoR, neomycin phosphotransferase gene; UTR, Untranslated region. GUS, β-glucuronidase gene, GFP, Green Fluorescence Protein gene, Nos, Nopaline synthase terminator, 35S, CAMV 35S gene, term, transcription terminator. Arrows indicate the direction of transcription, RB and LB, Right and Left border sequence, respectively [file 122_2023_4340_MOESM2_ESM.tif]

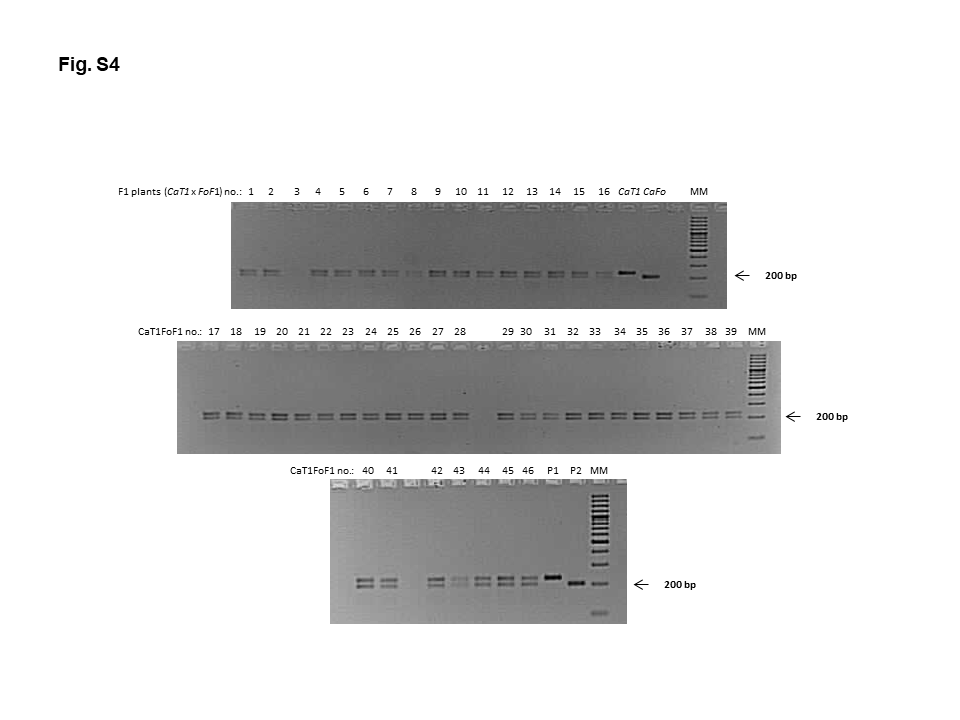

Supplement: Supplementary file 4 — Fig. S4 Demonstration of the hybrid nature of the F1 plants from cross CaT1 (♀) x CaFo (♂) using molecular marker M_472g4-OP. Fragment length polymorphism was detected after PCR amplification and agarose gel electrophoresis. Total DNA isolated from leaves of the F1 plants were amplified with primers Pr_472g4-OP F and Pr_472g4-OP R. Amplification products were separated in 3% agarose gel, and visualized bands were converted to genotypes. Upper and lower fragments were amplified from Bs5 and bs5 alleles, respectively. MM, 100 bp molecular ladder (Fermentas). F1 hybrid plants are numbered from 1- 46. CaT1, Ca var. T1; CaFo, Ca cv. Feherozon [file 122_2023_4340_MOESM4_ESM.tif]
