# Supplementary material for: The bs5 allele of the susceptibility gene Bs5 of pepper (Capsicum annuum L.) encoding a natural deletion variant of a CYSTM protein conditions resistance to bacterial spot disease caused by Xanthomonas species
Source: Theor Appl Genet. 2023 Mar 21;136(3):64. doi: 10.1007/s00122-023-04340-y (PMC10030403; doi:10.1007/s00122-023-04340-y)
Supplement: Supplementary file 3 — Fig. S3 N-terminal GFP-Bs5, GFP-bs5 constructs used for confocal microscopy. The double stranded cDNA sequence of Bs5 and bs5 was amplified from single stranded cDNA synthetized from polyA+ RNA isolated from young leaves of CaFo and CaT1 parental plants, respectively. For amplification, primer pairs Pr_Bs5c F7, Pr_Bs5c R7, and Pr_Bs5c F7, Pr_bs5 R7T, respectively, were used. Isolation of polyA + RNA and cDNA synthesis were carried out with Promega SV total RNA Kit and RevertAid First Strand cDNA Synthesis Kit. Double stranded cDNA sequences of CaFo-Bs5 and CaT1-bs5 were cloned into pGem-T Easy vector. From these constructs, CaFo Bs5 and CaT1 bs5 cDNA sequences were cloned in frame after the structural gene of green fluorescent protein (GFP) of pCambia-2302 vector using NheI, SalI and NheI, BstEII restriction enzymes (SalI and BstEII sticky ends were made blunt ended by Mung Bean nuclease. Panel A and B shows the functional and relevant restriction map of pCambia-2302 CaFo Bs5 and pCambia-2302 CaT1 bs5, respectively. The nucleotide and amino acid (AA) sequences of the junctions at the GFP – Bs5, and GFP—bs5 genes/proteins are shown in Panel C and D, respectively. Primer sequences are underlined. [file 122_2023_4340_MOESM3_ESM.pdf]

A

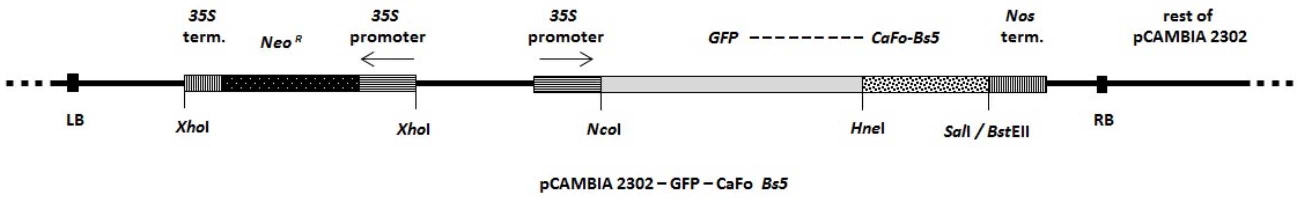

B

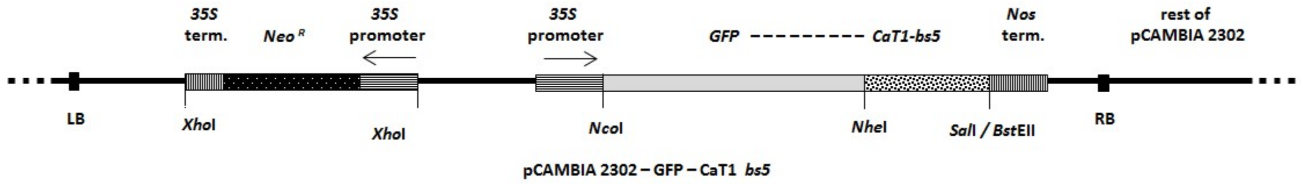

C

```
           NheI      Pr_Bs5c F7                                                                                      SalI/BstEII
  5'- GFP -  GCTAGCATGAGTTACTACAATCAACAACA - CaFo Bs5 cDNA - TGCTGTCTCTTGGATGCATGCTTTtgacacgtgaatcactagtgaattcgcggccgcctgcagg/c - Nos term.
AA sequence:  A  S  M  S  Y  Y  N  Q  Q                           3'-GAACCTACGTACGAAAACTGTGCAC - 5'     Pr_Bs5c R7
AA sequence:                                                    C  C  L  L  D  A  C  F  *
```

D

```
         NheI            Pr_Bs5c F7                                                                     SalI/BstEII
  5'- GFP -  GCTAGCATGAGTTACTACAATCAACAA - CaT1 bs5 cDNA - TGCTGTGATGCATGCTTTtgagtgaatcactagtgaattcgcggccgcctgcagg/c - Nos term.
AA  sequence: A  S  M  S  Y  Y  N  Q  Q                3'-   ACACTACGTACGAAAACT - 5'     Pr_bs5c R7T
AA  sequence:                                          C  C  D  A  C  F  *
```
