# Supplementary material for: The bs5 allele of the susceptibility gene Bs5 of pepper (Capsicum annuum L.) encoding a natural deletion variant of a CYSTM protein conditions resistance to bacterial spot disease caused by Xanthomonas species
Source: Theor Appl Genet. 2023 Mar 21;136(3):64. doi: 10.1007/s00122-023-04340-y (PMC10030403; doi:10.1007/s00122-023-04340-y)
Supplement: Supplementary file 5 — Fig. S5 Alignment of the nucleotide sequence of the Bs5 and bs5 loci. The sequence of the bs5 locus was determined by sequencing EcoRI and HindIII subclones as well as by high throughput SOLiD™ sequencing of BAC-50b4. The wild type Bs5 locus was sequenced from amplified genomic DNA of plant CaFo using specific primer pairs. These primer pairs were designed in such a way that the amplified fragments were overlapped with each other. The sequences of the bs5 and the Bs5 loci were deposited to the GenBank (GenBank accession no.: OM681616; GenBank accession no.: OM681615, respectively). The sequence of Bs5-TC, CaWD40-TC and primers for markers M_P6, M_WD40, M_bs5g, M_Bs5g were also aligned to the genomic sequence. CaBs5-TC and CaWD40-TC sequences were presented by red characters. Start and Stop codons were underlined. 5′-UTR/3′-UTR and coding regions were highlighted by lower and upper case letters, respectively. Introns and missing nucleotides (deletions) are indicated by dashes. The direction of primers is indicated by black arrows and by marking the 5′ end. Nucleotide alterations and indels in the sequence of bs5 gene were highlighted by turquoise-blue background color. poly(A), Polyadenylation tail [file 122_2023_4340_MOESM5_ESM.pdf]

Bs5 locus TTCGTGAGTATTATTCCTTTTTATTACTATAAAATTATTTTATTTTTATTATATTATACATATGGAATCTTTTCCAAAGTTGTTACGGGGAAATAGTGAAGTAACCGCGTGCCATGCGCGGAAAAGTGCTAAAATTAGTAAAAAAAATAATAATTATTTATTTATTTGATTGAATTAAAAATTATTTTAGTTATAATCAT 200
bs5 locus CGTGAGTATTATTCCTTTTTATTACTATAAAATTATTTTATTTTTATTATATTATACATATGGAATCTTTTCCAAAGTTGTTACGGGGAAATAGTGAAGTAACCGCGTGCCATGCGCGGAAAAGTGCTAAAATTAGTAACAAAAATAATAATTATTTATTTATTTGATTGAATTAAAAATTATTTTAGTTATAATCAT 200
Pr_P6 F 5′-TTTCGTGAGTATTATTCCTTTTTA →

Bs5 locus TTAGTAGAAGGGCAAAACGGAAAGAATTCAAATTTAAATCATTTGATTCGCAATTGTTTGATGAGAATACTCAATGTACAGAATTTTAAATGTAGTCGAGTTGATAGTGGAGTGATAGTTATTGTTCGAACTTCTAAGTGCATTCTGATGGGGCGAATGATCATTCTAGTTGTCTTTAATTAAAGTCGAGCACGCATGTT 400
bs5 locus TTAGTAGAAGGGCAAAACGGAAAGAATTCAAATTTAAATCATTTGATTCGCAATTGTTTGATGAGAATACTCAATGTACAGAATTTTAAATGTAGTCGAGTTGATAGTGGAGTGATAGTTATTGTTCGAACTTCTAAGTGCATTCTGATGGGGCGAATGATCATTCTAGTTGTCTTTAATTAAAGTCGAGCACGCATGTT 400

Bs5 locus CTCAAGCGGTATTAATGGGGCACTAGGCTTGGTTTGTCTATTTATGGATGGAGAGTGATGCTAAGGTTCAGTTGTGTGTTCGAATTCTTTCGTATAAGTTAAACAACTTTATTTGAAATGATGAATATTAAAGTCTCATGCAAGCGACCAATCTCCTTTTATGTATAAGACAATAAATTCTTTTGTTGTGCAAGTGGACT 600
bs5 locus CTCAAGCGGTATTAATGGGGCACTAGGCTTGGTTTGTCTATTTATGGATGGAGAGTGATGCTAAGGTTCAGTTGTGTGTTCGAATTCTTTCGTATAAGTTAAACAACTTTATTTGAAATGATGAATATTAAAGTCTCATGCAAGCGACCAATCTCCTTTTATGTATAAGACAATAAATTCTTTTGTTGTGCAAGTGGACT 600

Bs5 locus TAATTGGTAAGGAGTGGTGGAGTTTCATTAAAAGGGCTATATAATAATTACTAATATACTGATATATGTATTTTTTTTCAAACATAGTCCTCAATTTCAAAGAAAAACATAGCGAAAAAGCAGCGCGAAGGGGAGTTTTTAACCTTATTACAATGCTTCCAAACATGAATAAATTTTTTGATTCATCAATCGACAACATC 800
bs5 locus TAATTGGTAAGGAGTGGTGGAGTTTCATTAAAAGGGCTATATAATAATTACTAATATACTGATATATGTATTTTTTTTCAAACATAGTCCTCAATTTCAAAGAAAAACATAGCGAAAAAGCAGCGCGAAGGGGAGTTTTTAACCTTATTGCAATGCTTCCAAACATGAATAAATTTTTTGATTCATCAATCGACAACATC 800
Pr_P6 R ← TGTATCGCTTTTTCGTCGC-..5′

Bs5 locus TAGTTTCGAGGAGAGGTATATTTAAGTCCAATATGTATGTTATTCATTTAAATGATCTCGTCATCTAATTTGTTTCACTTTATATTAGTGCATTTGAGAATTGTTGTAGAATTGCTCTTGAAGTTATAGGAGCAATTATGAATGAAACTGAAAGAGATAAGAGTTGAAGTAAAACTCTTCGTCTCATTGAAAATGTATGG 1000
bs5 locus TAGTTTCGAGGAGAGGTATATTTAAGTCCAATATGTATGTTATTCATTTAAATGATCTCGTCATCTAATTTGTTTCACTTTATATTAGTGCATTTGAGAATTGTTGTAGAATTGCTCTTGAAGTTATAGGAGCAATTATGAATGAAACTGAAAGAGATAAGAGTTGAAGTAAAACTCTTCGTCTCATTGAAAATGTATGG 1000

Bs5 locus AAACAAAAAAAGATATATATCTTCATGTGTTCGAGTCAAAGGACGAACTTCTAATAATTGCCTTCAATTTATAAAGTCATTTGAAGGGAAACTTCTTGTTTCGGGTGGCTATAATACAAATGAGAGAGATGTGGCTATTTCTTAAAGTTGTGCAGATTTTGTCTCATTTGGACATTTATTTTGGGCCAAATATGGTTTGC 1200
bs5 locus AAACAAAAAAAGATATATATCTTCATGTGTTCGAGTCAAAGGACGAACTTCTAATAATTGCCTTCAATTTATAAGGTCATTTGAAGGGAAACTTCTTGTTTCGGGTGGCTATAATACAAATGAGAGAGATGTGGCTATTTCTTAAAGTTGTGCAGATTTTGTCTCATTTGGACATTTATTTTGGGC-AAATATGGTTTGC 1200

Bs5 locus CCAGATTAATGGGCTATTGGAAACAAGCTTCAAAGTAAAATAACATAAACATACATCTTAACTTACTATATTTATACAAATTTTCATCCTTACAAAGCAATTACAAGCTAATTATATATCTTTCTTTCTTTCTTTTTCGTTTTCCACCCAA-TTTGATACCCTCATTGACCCCCCTCCCCCCCGACTAATTCGGATCGC 1400
bs5 locus CCAGATTAATGGGCTATTGGAAACAAGCTTCAAAGTAAAATAACATAAACATACATCTTAACTTACTATATTTATACAAATTTTCATCCTTACAAAGCAATTACAAGCTAATTATATATCTTTCTTTCTTTCTTTTTCGTTTTCCACCCAATTTTGATACCCTCATTGACCCCCCTCCCCCCCGACTAATTCGGATCGC 1400

Bs5 locus ACACTGCAGGACTCATTCTGGTGGCGCTCCCATCAGGAGCTAATTATGTATCTTTATAAAGACAAAAAATATATTTTTGTTAGATGTATTATGTATCTTGATAAATCCGACATCGAAATAAATGTATCAGGAGTTAATTATGTATCTTTACAAAGAAAAAATACATCTTCATTGGATGTATCGGAAGCTAATTATGTATC 1600
bs5 locus ACACTGCAGGACTCATTCTGGTGGCGCTCCCATCAGGAGCTAATTATGTATCTTTATAAAGACAAAAAATATATTTTTGTTAGATGTATTATGTATCTTGATAAATCCGACATCGAAATAAATGTATCAGGAGTTAATTATGTATCTTTACAAAGAAAAAATACATCTTCATTGGATGTATCGGAAGCTAATTATGTATC 1600

Bs5 locus TCAGCAAATTCAAAATTGAAATTTCAATAATTATGTAAAATATCAAAACAAAAATTATAATTATGCTTCAAACTGTCGAAATTTATATTATTTTTACTAATTTCAAAAGATACGAATAATCTGAAAGGTTCTTGTACTTGTTCTGATTTATTATTCTGACTCCTTTATTTTGTTTTCTAAATAGAAGGCAAAACTTTTTT 1800
bs5 locus TCAGCAAATTCAAAATTGAAATTTCAATAATTATGTAAAATATCAAAACAAAAATTATAATTATGCTTCAAACTGTCGAAATTTATATTATTTTTACTAATTTCAAAAGATACGAATAATCTGAAAGGTTCTTGTACTTGTTCTGATTTATTATTCTGACTCCTTTATTTTGTTTTCTAAATAGAAGGCAAAACTTTTTT 1800

Bs5 locus TTTTTTTT--GTGATTATTGCCGTTTATTTATTTATTTGGTAGAATAAGTGATGATGATTTTACAAGCATTTTGCATGTTAAGATGTAATTTTTCTGGACTTTTTAATTTCGATTAATTGAATCTATTGATATGGGACTTCTCAATGTATTCTTCTACATCCATTTTCTAATTTTCAGATGTATGAAATTATGTATTCTC 2000
bs5 locus TTTTTTTTTTGTGATTATTGCCGTTTATTTATTTATTTGGTAGAATAAGTGATGATGATTTTACAAGCATTTTGCATGTTAAGATGTAATTTTTCTGGACTTTTTAATTTCGATTAATTGAATCTATTGATATGGGACTTCTCAATGTATTCTTCTACATCCATTTTCTAATTTTCAGATGTATGAAATTATGTATTCTC 2000

Bs5 locus TAAATAATAGAAAAAAAATCAAATCAAATCAAAATCGAATTCAAACAACCACATCAAATTTCAAAGATCGAACATCATTGCATATCTATCATTGTCACTTCCATCATCTCCATTGAAATCTCTTTCCGAAAGAGAAGATGGAAAAGATTTCAATGGAGAAGATAGAAAGTGAAGATGCTCGACACACAACTTCTTGTATT 2200
bs5 locus TAAATAATAGAAAAAAAATCAAATCAAATCAAAATCGAATTCAAACAACCACATCAAATTTCAAAGATCGAACATCATTGCATATCTATCATTGTCACTTCCATCATCTCCATTGAAATCTCTTTCCGAAAGAGAAGATGGAAAAGATTTCAATGGAGAAGATAGAAAGTGAAGATGCTCGACACACAACTTCTTGTATT 2200

Bs5 locus CAGAATTCACTGACTCGATTAATATGTCTTATATAAAATACGCTTTAGCTGACAAATCTACGACTAACAAACGAAGATGGGTGATCTATTTATACTATATCAAATGTCACAAAAAGATTAATCTCATTAAATATTTCATTTGGAAAATCAGATTTGCTACCATTTCTCATAAAAGTATACCAATATTTTTTAGTCTAAAT 2400
bs5 locus CAGAATTCACTGACTCGATTAATATGTCTTATATAAAATACGCTTTAGCTGACAAATCTACGACTAACAAACGAAGATGGGTGATCTATTTATACTATATCAAATGTCACAAAAAGATTAATCTCATTAAATATTTCATTTGGAAAATCAGATTTGCTACCATTTCTCATAAAAGTATACCAATATTTTTTAGTCTAAAT 2400

Bs5 locus TATTAATGAAACATGGTGTCCAATTAAATGTGATACCAAATGTTAATAGGTAAGTGTTTAAATGTACTTACCAAGTATATAAAAAATTGGTATTTAATTACAAATGCAATCTGTATCAACAATACAAAATAAAAAAATAATATAATTGATTATTTGAAGTGGAATTGGTACATATAAGATGAATAAAATAGACTATTACA 2600
bs5 locus TATTAATGAAACATGGTGTCCAATTAAATGTGATACCAAATGTTAATAGGTAAGTGTTTAAATGTACTTACCAAGTATATAAAAAATTGGTATTTAATTACAAATGCAATCTGTATCAACAATACAAAATAAAAAAATAATATAATTGATTATTTGAAGTGGAATTGGTACATATAAGATGAATAAAATAGACTATTACA 2600

Bs5 locus ACTATTTAATTAATTATAAAAAGACCAATTTGCAATTAAAAATTTGGAACACAACTGTCTAAGAAAATTTTCCCAACTATGATAAAAAAAAAAAAAAAAAAAGACAGCAAAACAATTGAAGTAGTCCATATGTTTGTTATTAATGAAAGTTTAGGAGGTTCAATTATGTCATAGTAGAATATTATTTAATTCCTTTCTTG 2800
bs5 locus ACTATTTAATTAATTATAAAAAGACCAATTTGCAATTAAAAATTTGGAACACAACTGTCTAAGAAAATTTTCCCAACTATGATAAAAAAAAAAAAAAAAAA-GACAGCAAAACAATTGAAGTAGTCCATATGTTTGTTATTAATGAAAGTTTAGGAGGTTCAATTATGTCATAGTAGAATATTATTTAATTCCTTTCTTG 2800

Bs5 locus TCACAAACTATTTAATGTGAGAACAAGCTAGAGTATTGAAAGACATTGAAAAATTTATTTTTAATTATGCTGAGAAGATTGAAAAAACATTTAATTGCAAAGTTTACCAAATGTGTCTCATTTTTCAACATTGGAGCTTCCTATGAAAAGTCTACTCCAACTTTCTTTCAATTAAATTAAATTTGGATTTATTTATTCTT 3000
bs5 locus TCACAAACTATTTAATGTGAGAACAAGCTAGAGTATTGAAAGACATTGAAAAATTTATTTTTAATTATGCTGAGAAGATTGAAAAAACATTTAATTGCAAAGTTTACCAAATGTGTCTCATTTTTCAACATTGGAGCTTCCTATGAAAAGTCTACTCCAACTTTCTTTCAATTAAATTAAATTTGGATTTATTTATTCTT 3000

Bs5 locus ATTTAAACATGAAAAGTATTCTTGGGGATTATTTTCACTTATTTTGATCATATATACTCATGAAAACATTAATTAGAACTCGGAACTAAATTTAAAACGTGATCAAAATAGGTCACCTATTAGGCTAAATGTAATAAATTATTACGTTTTTCACTTTTTTCTTAACGATCAACATGATTTGTAAAACGTAATAATTTCGT 3200
bs5 locus ATTTAAGCATGAAAAGTATTCTTGGGGATTATTTTCACTTATTTTGATCATATATACTCATGAAAACATTAATTAGAACTCGGAACTAAATTTAAAACGTGATCAAAATAGGTCACCTATTAGGCTAAATGTAATAAATTATTACGTTTTTCACTTTTTTCTTAACGATCAACATGATTTGTAAAACGTAATAATTTCGT 3200

Bs5 locus ATAATCTTATTCGATTATCGATTTATTCAATAACCCAATAGAAAAATTAAAACCAAATCAATAACCCAATAATTTTTTAAAAAAATCATTAACCCGATAAACATAAATCAATAACATTTTTATCGATTCGATTTTTT-ACACCGGTAACTTAAATTGTGATTTCTTACTCCATTAATTGTAATAAAAAACGTATTTGTCT 3400
bs5 locus ATAATCTTATTCGATTATCGATTTATTCAATAACCCAATAGAAAAATTAAAACCAAATCAATAACCCAATAATTTTTTAAAAAAATCATTAACCCGATAAACATAAATCAATAACATTTTTATCGATTCGATTTTTTTACACCGGTAACTTAAATTGTGATTTCTTACTCCATTAATTGTAATAAAAAACGTATTTGTCT 3400

Bs5 locus ATGTGAGTCTTTTCAAAATCTTAATCGAAACACTAATCAGTAGCTAATATCAAAAAAGGGACAGGGTTGGGGGTGGGGTGGAAGGATTTTACGTGGGGTGGGGTGGGGTGGGAGGGTTGTACGTGGGGTGGGTTAAATTTTAAAGTGAGAAAAGTAAAGGTGATTTGATGTGGGCAACTGTACACTGTTTGTAGTTTGTA 3600
bs5 locus ATGTGAGTCTTTTCAAAATCTTAATCGAAACACTAATCAGTAGCTAATATCAAAAAAGGGACAGGGTTGGGGGTGGGGTGGAAGGATTTTACGTGGGGTGGGGTGGGGTGGGAGGGTTGTACGTGGGGTGGGTTAAATTTTAAAGTGAGAAAAGTAAAGGTGATATGATGTGGGCAACTGTACACTGTTTGTAGTTTGTA 3600

Bs5 locus ATAATTTATTACGTTTTATAAGCAGTGCTTGTAAAACGTAATAATTTATTACAAACTACATGATTTGACATAAAGAAGATATGATAGCACACTGTTTTTTTTGTGGAAAATGTAATAAATTATTACGTTTTACAGACCGTGTTGATTGTTAAGAAAAAAGTGAAAGACGTAATAATTTATTACATTTAGCCTATTTTGGT 3800
bs5 locus ATAATTTATTACGTTTTATAAGCAGTGCTTGTAAAACGTAATAATTTATTACAAACTACATGATTTGACATAAAGAAGATATGATAGCACACTGTTTTTTTTGTGGAAAATGTAATAAATTATTACGTTTTACAGACCGTGTTGATTGTTAAGAAAAAAGTGAAAGACGTAATAATTTATTACATTTAGCCTATTTTGGT 3800

Bs5 locus AACTTTTTAAATAGGTGGCCTGTTTTGATCACTTTTTATTTTTATTTTATGACTTATTTTGGTTTCGAGTTCTATTAATTACCCCTAACTACGTTGCGACAATTTTTTTGGGTCCTTTTTACATCTCTTTGACCATAAAATAATATAAGATATTACTCCCTCTGTCTTAAATTACCCATTCCAAATTGAAATGACACTCT 4000
bs5 locus AACTTTTTAAATAGGTGGCCTGTTTTGATCACTTTTTATTTTTATTTTATGACTTATTTTGGTTTCGAGTTCTATTAATTACCCCTAACTACGTTGCGACAATTTTTTTGGGTCCTTTTTACATCTCTTTGACCATAAAATAATATAAGATATTACTCCCTCTGTCTTAAATTACCCATTCCAAATTGAAATGACACTCT 4000

Bs5 locus GATTAAGAAAAATAATTAATAACATGGCTAGTTTACCATAGTACTCCTATTAAATAATGTTTACATTTTAATTTGAAGAAAAAATAATTAATGCAAAGGGTAAAATATGAAAAAAAAAATATCTCTTCTTGATTAATCAATAAAATGAGAAATCAAATTAAAAAAATTTGGGTCTAATAATTTGGGACGGAGAAAGTATTT 4200
bs5 locus GATTAAGAAAAATAATTAATAACATGGCTAGTTTACCATAGTACTCCTATTAAATAATGTTTACATTTTAATTTGAAGAAAAAATAATTAATGCAAAGGGTAAAATATGAAAAAAAAAATATCTCTTCTTGATTAATCAATAAAATGAGAAATCAAATTAAAAAAATTTGGGTCTAATAATTTGGGACGGAGAAAGTATTT 4200

Bs5 locus GATATACGAGATAGGATGCAAGGATATCCCATCGAATTATCTTATCTCACCTGTCATATGGATATTCCATCATTTCAATATAAACCATGGGATAAAACAGTCTCCAAATTAATTAGGACTTCACATCTAATATGAGATAATGTTAATTTCAAATTTAATCTTCGATTAGTTTAAAGCTAAGAGTAATCCTTCTATTCTCA 4400
bs5 locus GATATACGAGATAGGATGCAAGGATATCCCATCGAATTATCTTATCTCACCTGTCATATGGATATTCCATCATTTCAATATAAACCATGGGATAAAACAGTCTCCAAATTAATTAGGACTTCACATCTAATATGAGATAATGTTAATTTCAAATTTAATCTTCGATTAGTTTAAAGCTAAGAGTAATCCTTCTATTCTCA 4400

Bs5 locus GAGAATAATTATCCTCTGTTTGCACAATCTAAACAAATGACCAAGTATTATTTACAAATTACAATGAATATAAACTAGTGATAGGAATAGCTTAATATCAAAAGAAACATTCATATTTTATTTTTCTATTCAACAATCTTGATACATTTTTCCTCTTATCTTGAGAAATAATTTTAGGAATTCAATCTTATTGCACATA 4600
bs5 locus GAGAATAATTATCCTCTGTTTGCACAATCTAAACAAATGACCAAGTATTATTTACAAATTACAATGAATATAAACTAGTGATAGGAATAGCTTAATATCAAAAGAAACATTCATATTTTATTTTTCTATTCAACAATCTTGATACATTTTTCCTCTTATCTTGAGAAATAATTTTAGGAATTCAATCTTATTGCACATA 4600
Pr_Bs5g F3 5′-TCTGCAGTGCACAATCTAAACAAATGACCA→

Bs5 locus CAATAAAATCTTACGATAATTTATAACGGAAAAAATAATTACTGTAAATTTTTTCCGTCGCTTCTTAACTTCTAATTGTGGATAAAAATAGTTTAAAATTTAATACTTCCTTCGTTTCAAAATAATTGAATTGTTGAGTATTTTTTAGGGTTCAAAATAATTAAATTGTTCATTATTCAAGATATATGTTGAATTTTTTT 4800
bs5 locus CAATAAAATCTTACGATAATTTATAACGGAAAAAATAATTACTGTAAATTTTTTCCGTCGCTTCTTAACTTCTAATTGTGGATAAAAATAGTTTAAAATTTAATACTTCCTTCGTTTCAAAATAATTGAATTGTTGAGTATTTTTTAGGGTTCAAAATAATTAAATTGTTCATTATTCAAGATATATGTTGAATTTTTTT 4800

Bs5 locus ATTTTTTTTTAAATTTACTTTTATTAATTAAATTTTCAAGATTGAGTTCCAATGGTCATTATTAATGTTTTAGAATTTGAAAAGGACAAAAATGAAAAAACATGACTAATTTATATTTTTATCTTTTTTTCTTAAAAGTGTGTCATATTTTAATAATTCAATTATTTTGAAACGAGGAGAGTAATTTTTTTTAATCAACT 5000
bs5 locus ATTTTTTTTTAAATTTACTTTTATTAATTAAATTTTCAAGATTGAGTTCCAATGGTCATTATTAATGTTTTAGAATTTGAAAAGGACAAAAATGAAAAAACATGACTAATTTATATTTTTATCTTTTTTTCTTAAAAGTGTGTCATATTTTAATAATTCAATTATTTTGAAACGAGGAGAGTAATTTTTTTTAATCAACT 5000

Bs5 locus AAAACCATAATTTTATACTCTCTTCGTCCCAAATTTTCTAATTTGTTTTTCCATGTGTTTACCCTTTGCATTATTTCTTTTTTCTTCAAATTAAAATGTAAACATGATTTAATAGGGATATTATGGTAAACTAGACATGTTATTAATTATTTTTCTTAATCAATGTGTCATCTCAATCTGAAACGGAGGGAGTATCTTTA 5200
bs5 locus AAAACCATAATTTTATACTCTCTTCGTCCCAAATTTTCTAATTTGTTTTTCCATGTGTTTACCCTTTGCATTATTTCTTTTTTCTTCAAATTAAAATGTAAACATGATTTAATAGGGATATTATGGTAAACTAGACATGTTATTAATTATTTTTCTTAATCAATGTGTCATCTCAATCTGAAACGGAGGGAGTATCTTTA 5200

Bs5 locus TCTTTTTTTTCTTAAAACTGTGTCATATTTCAACAATTCAATTATTTTAAAACGGAGGAATTAATTTTTTTCAATCAAATAAAACCATAATTTTATAATAATTCTTTAAAAAAAAAATAAAAAAGAATTTCCGCGCATTGGACGCGGGTACGTATTAAAGCTACCTATGACAACATGGGAAAAGATTACATTATAAAAAAA 5400
bs5 locus TCTTTTTTTTCTTAAAACTGTGTCATATTTCAACAATTCAATTATTTTAAAACGGAGGAATTAATTTTTTTCAATCAAATAAAACCATAATTTTATAATAATTCTTTAAAAAAAAAATAAAAAAGAATTTCCGCGCATTGGACGCGGGTACGTATTAAAGCTACCTATGACAACATGGGAAAAGATTACATTATAAAAAAA 5400
Ca_CYSTM1-TC

Bs5 locus ACAAAAATAAGAGTTTCTTGGAATGTGCAATCGTCTTTGTTTTCCCCTTTGACTTTACTCTATAAAAACTTCACAAATATCACCTCTTCACTGTACCCCATTATCTTTCTTTGTGGTTAAGCAAATACACAAAATAAATAAATATAACTCTCCTCTTAGATTAAACTAGTAGATCCATCAACAATGAGTTACTACAATCA 5600
bs5 locus ACAAAAATAAGAGTTTCTTGGAATGTGCAATCGTCTTTGTTTTCCCCTTTGACTTTACTCTATAAAAACTTCACAAATATCACCTCTTCACTGTACCCCATTATCTTTCTTTGTGGTTAAGCAAATACACAAAATAAATAAATATAACTCTCCTCTTAGATTAAACTAGTAGATCCATCAACAATGAGTTACTACAATCA 5600
Ca_CYSTM1-TC cccctttgactttactctataaaaacttcacaaatatcacctcttcactgtaccccattatctttctttgtggttaagcaaatacacaaaataaataaatataactctcctcttagattaaactagtagatccatcaacaATGAGTTACTACAATCA

Bs5 locus ACAACAACCTCCTGTTGGTGTACCTCCACCACAAGGTAAAAAAAAAAAAAGGAAGAAACAACTCTGACTTTGTTGTATGTGAATTGTTTTAGTTATTAGATCTGATTGATTTTTATTTTTTTGGGGGTATTTTTTGTGATTTAGGGTATCCACCAGAAGGTTACCCAAAAGATTCATACCCACCACCTGGATATCCACAG 5800
bs5 locus ACAACAACCTCCTGTTGGTGTACCTCCACCACAAGGTAAAAAAAAAAAAAGGAAGAAACAACTCTGACTTTGTTGTATGTGAATTGTTTTAGTTATTAGATCTGATTGATTTTTATTTTTTTGGGGGTATTTTTTGTGATTTAGGGTATCCACCAGAAGGTTACCCAAAAGATTCATACCCACCACCTGGATATCCACAG 5800
Ca_CYSTM1-TC ACAACAACCTCCTGTTGGTGTACCTCCACCACAAG---GGTATCCACCAGAAGGTTACCCAAAAGATTCATACCCACCACCTGGATATCCACAG

Bs5 locus CAAGGGTACCCTCAACAAGGGTATCCACCACAAGGGTACCCTCCACAGTATGCACCTCAGTATGGTGCACCACCTCCTCAACAACAACATCAATCATCTAGTAGTACTGGATTATTGCAAGGATGGTATGTACACCTTATTAGGACTCTATTTTTTTATATGTTGACTTGTATGGATGTGTTATGTAGGATCTGAGGTTT 6000
bs5 locus CAAGGGTACCCTCAACAAGGGTATCCACCACAAGGGTACCCTCCACAGTATGCACCTCAGTATGGTGCACCACCTCCTCAACAACAACATCAATCATCTAGTAGTACTGGATTATTGCAAGGATGGTATGTACACCTTATTAGGACTCTATTTTTCTATATGTTGACTTGTATGGATGTGTTATGTAGGATCTGAGGTTT 6000
Ca_CYSTM1-TC CAAGGGTACCCTCAACAAGGGTATCCACCACAAGGGTACCCTCCACAGTATGCACCTCAGTATGGTGCACCACCTCCTCAACAACAACATCAATCATCTAGTAGTACTGGATTATTGCAAGGATG---

Bs5 locus ATAGGATTGAGTTGACCATGTCTTGTTTGTGTGATAAGATATGAATTGATGTGAACTTGATTCATCAAGATCTATATGACCTCAGGTTTTGTTGACTGAGTTGCCTGAATTTTTTATCAAAATATGTATTAGGTTTTAAAAAGATTTAACGGATCAAAGCGGTGTTAAAACTAGAAATAGTTTATGACAGTAACGTTAAC 6200
bs5 locus ATAGGATTGAGTTGACCATGTCTTGTTTGTGTGATAAGATATGAATTGATGTGAACTTGATTCATCAAGATCTATATGACCTCAGGTTTTGTTGACTGAGTTGCCTGAATTTTTTATCAAAATATGTATTAGGTTTTAAAAAGATTTAACGGATCAAAGCGGTGTTAAAACTAGAAATAGTTTATGACAGTAACGTTAAC 6200
Ca_CYSTM1-TC --

Bs5 locus AAGACTCTGCAACTCTGCCACATCATACTATTTGTCAGAGTTGCAGAGTCTCTGAAGTTCTAAGTCAACTCTGCAACATCAGACTCACAAAGTTGATTTGGCAGAATTGCAGTGTCTCTTCATTATTGGTGAGGTTTTAAGTCAAAAACGTTATTGTCAGTAACGATTCCTGCTTTGACGTCGTCCATTGTTCATTTTGT 6400
bs5 locus AAGACTCTGCAACTCTGCCACATCATACTATTTGTCAGAGTTGCAGAGTCTCTGAAGTTCTAAGTCAACTCTGCAACATCAGACTCACAAAGTTGATTTGGCAGAATTGCAGTGTCTCTTCATTATTGGTGAGGTTTTAAGTCAAAAACGTTATTGTCAGTAACGATTCCTGCTTTGACGTCGTCCATTGTTCATTTTGT 6400
Ca_CYSTM1-TC --

Bs5 locus GTCCGTTAATTTTTTTAAAACTTAATAACGTTTATACTGGTTTTGGAAAGTTTTCTAAAAACATACTACTCTGGTGAATTGATTTAAAAAATAATTTATTTTGGTCAAAACTTCAAGTTGCTTGGTGAAGCTGACCTCGTGTCTAACCGGGAGGTACTGGCTGAAAATAGCCTCATGCAAGATAAGGCTAGGTTATAATA 6600
bs5 locus GTCCGTTAATTTTTTTAAAACTTAATAACGTTTATACTGGTTTTGGAAAGTTTTCTAAAAACATACTACTCTGGTGAATTGATTTAAAAAATAATTTATTTTGGTCAAAACTTCAAGTTGCTTGGTGAAGCTGACCTCGTGTCTAACCGGGAGGTACTGGCTGAAAATAGCCTCATGCAAGATAAGGCTAGGTTATAATA 6600
Ca_CYSTM1-TC --

Bs5 locus AACCTTTGTGGTTCGGTTCTTCCTACACACCGAGTCTCATGCTGTTTCCAATTATCGGTCGGATTAATAATCGATTTTTTTTATTTTATTTTTTTTTCAGTTTGGCTGCTCTTTGCTGTTGCTGTCTCTTGGATGCATGCTTTTGATGCTGTAAATGATCTGTACGCAAAGTGTTGATGACAAAAGATGATTGAAATCCAT 6800
bs5 locus AACCTTTGTGGTTCGGTTCTTCCTACACACCGAGTCTCATGCTGTTTCCAATTATTGGTCGGATTAATAATCGATTTTTTTTATTTTATTTTTTTTTCAGTTTGGCTGCTCTTTGCTGTTGCTGT------GATGCATGCTTTTGATGCTGTAAATGATCTGTACGCAAAGTGTTGATGACAAAAGATGATTGAAATCCAT 6800
Ca_CYSTM1-TC ---TTTGGCTGCTCTTTGCTGTTGCTGTCTCTTGGATGCATGCTTTtgatgctgtaaatgatctgtacgcaaagtgttgatgacaaaagatgattgaaatccat
Pr_bs5g F1 5′-CTGCTCTTTGCTGTTGCT →

Bs5 locus TATCATAGTCTAGATTATTTTCCTTGAACGTGTTTTGTCCTTGTTGTCCTGTCATTTATAAATAATTTGATCTTGCTATGGTGTCTATTTGCCAAATTATAGGTTTATGTACAACGTGAGAGATTGTATTTTATTTTTTATGTTTTGGACCTCAATATGTGAATCAATGCACCTTGATTTGGTTAAACAATTTATCGCCT 7000
bs5 locus TATCATAGTCTAGATTATTTTCCTTGTATGTGTTTTGTCCTTGTTGTCCTGTCATTTATAAATAATTTGATCTTGCTATGGTGTCTATTTGCCAAATTATACGTTTATGTACAACGTGAGAGATTGTATTTTATTTTTTATGTTTTGGACCTCAATATGTGAATCAATGCACCTTGATTTGGTTAAACAATTTATCGCCT 7000
Ca_CYSTM1-TC tatcatagtctagattattttccttgaacgtgttttgtccttgttgtcctgtcatttataaataatttgatcttgctatggtgtctatttgccaaattataggtttatgtacaacgtgagagattgtattttattttttatgttttggacctcaatatgtgaatcaatgcaccttgatttggttaaa-poly(A.
Pr_bs5g R1 ← CGATACCACAGATAAACGG-5′.

Bs5 locus CAT--GTGTCTATAATCCAAGCGCTTGGATAGTGGCGGATTCAGGATTTACTTTGAGAGGGTTCAGAAGTATATATACGAGAATTAATCAAAGGGGTTTAATATCTATTATATGTGCATTAAAATATCTGTAATCATGTAAAAATAGTATAAAAACAGTACTCGCTTCGTTTAAAAAAAAAATGATTTACTTTTCTTTTT 7200
bs5 locus CATGTGTGTCTATAATCCAAGCGCTTGGATAGTGGCGGATTCAGGATTTACTTTGAGAGGGTTCAGAAGTATATATACGAGAATTAATCAAAGGGGTTTAATATCTATTATATGTGCATTAAAATATCTGTAATCATGTAAAAATAGTATAAAAACAGTACTCGCTTCGTTTAAAAAAAAA-TGATTTACTTTTCTTTTT 7200

Bs5 locus AGTTTGTTTAAAAAAAAAATGATTCATTTCCTTTTTATTCACTTTTTATTCTAATTTTTCACATGGTATATTTAAGACCACAAGATTGAAGGGCATTTTGGTACATTTGACATATCTTTAATTTAACACTACAAGATTCAAAAGTCTTTTTTATTTTCTTAAACTCCGTGTTTGGTCAAACTAGATCATTTTTTTTTAA 7400
bs5 locus AGTTTGTTT-AAAAAAAAATGATTCATTTCCTTTTTATTCACTTTTTATTCTAATTTTTCACATGGTATATTTAAGACCACAAGATTGAAGGACATTTTGGTACATTTGACATATCTTTAATTTAACACTACAAGATTCAAAAGTCTTTTTTATTTTCTTAAACTCCGTGTTTGGTCAAACTAGATCATTTTTTTTTAA 7400

Bs5 locus ACGGAGGGAGTATATTTTTTAGCCGAAGGGGGTTCGGATGAACTCCTGGTGCAAAATGGCTTCGCCATTGCTCTAGACGTTGCTGATTTGTATTTAATGTGTGGTGAATACAACCTTTGGGTCAAGAGTCGGTGGTTCGTCGTATTTATAGTCTATTTGGTCAAGCTTTACAACAACAATAAACCCAGTGTATTCCACAT 7600
bs5 locus ACGGAGGGAGTATATTTTTTAGCCGAAGGGGGTTCGGATGAACTCCTGGTGCAAAATGGCTTCGCCATTGCTCTAGACGTTGCTGATTTGTATTTAATGTGTGGCGAATACAACCTTTGGGTCAAGAGTCGGTGGTTTGTCGTATTTATAGTCTATTTGGTCAAGTTTTACAACAACAATAAATCCAGTGTATTCCACAT 7600
Pr_Bs5g R3 ← GCCTACTTGAGGACCACGTTGACGTCA-5′.

Bs5 locus AGTGGGGTCTGGAAAGGGTAAAATGCATGTAGTTCATACCACTATCTTCGGAAAAGTAGAAAGGCTGTTTCCGATAGACCCCGGCTCAAGCTTTGAAGTCGTTTATTTGAAGGTAATTTTTGTGATAAGCGCTGTTCGGAAAAGATATATTTTTCGCTTTTGAAAAATAGTTTCGACTATTATTCAAAAATATTTATCTA 7800
bs5 locus AGTGGGGTCTGGAAAGGGTAAAATGCATGTAGTTCATACCACTATCTTCGGAAAAGTAGAAAGGCTGTTTCCGATAGACCCCGGCTCAAGCTTTGAAGTCGTTTATTTGAAGGTAATTTTTGTGATAAGCGCTGTTCGGAAAAGATATATTTTTCGCTTTTGAAAAATAGTTTCGACTATTATTCAAAAATATTTATCTA 7800

Bs5 locus AAAGTTTTAGCTACACACGTTTTAACTTTTAAAAGTTATGCAAAGCACATGAAATGTCTTAAAGTTGGCCAAAGAGGCTTATAATTATGCCACTATTCATGAACATGAGGGTTCATAAAAATGAGGATTCATAAAATTCATCGTATATATTTGAATTTGGTGGGGTGTCATAAGAAAAGAAAAAAGTTGAATTAGGTGTA 8000
bs5 locus AAAGTTTTAGCTACACACGTTTTAACTTTTAAAAGTTATGCAAAGCACATGAAATGTCTTAAAGTTGGCCAAAGAGGCTTATAATTATGCCACTATTCATGAACATGAGGGTTCATAAAAATGAGGATTCATAAAATTCATCGTATATATTTGAATTTGGTGGGGTGTCATAAGAAAAGAAAAAAGTTGAATTAGGTGTA 8000

Bs5 locus CTTAAATGGCTTAAAATTATAAGCATGACAAGATGGGAAAATTGTAATTGAGTTCAAATGTTGGTGTTGTCCACCTGAGGATGTTAAATGGGCAGGACCATAAAACCTTT----------------TTGCTAATTAGCTCATGAAAATATGATCAAACTCTTTTTAGTTTATACATGACTTTCTTTTAGTCCGTTTTAAC 8200
bs5 locus CTTAAATGGCTTAAAATTATAAGCATGACAAGATGGGAAAATTGTAATTGAGTTCAAATGTTGGTGTTGTTCACCTGAGGATGTTAAATGGGCAGGACCATAAAACCTTTTTGGGGTAAAGCTTCGTTGCTAATTAGCTCATGAAAATATGATCAAACTCTTTTTAGTTTATACATGACTTTCTTTTAGTCCGTTTTAAC 8200

Bs5 locus TTTTAATCAAACTTTTTTCATTCAAATAACTTTTGATCGATCAATAATTCATTCATTTATTAATCTAATCCATTTTAATCTGTTCAAATCCAACCTTATCTACCCATTTGACACCTCTAACATGCTCCACTTTCAATCTCATTATCATCCCAAACAATATTGTACTATATTAGTGTAGACATCGAAATCTTGTAAAACTC 8400
bs5 locus TTTTAATCAAACTTTTTTCATTCAAATAACTTTTGATCGATCAATAATTCGTTCATTTATTAATCTAATCCATTTTAATCTGTTCAAATCCAACCTTATCTACCCATTTGACACCTCTAACATGCTCCACTTTCAATCTCATTATCATCCCAAACAATATTGTACTATATTAGTGTAGACATCGAAATCTTGTAAAACTC 8400

Bs5 locus TACAAGAATTCAAATTCTATTGGAGTTCTTGTAGGCTATCCTAATCCTAAATTATGTCTATAAAAAGAATGGTTTAATCTTGGAAAAATCATCTTGAATATCCCATAAACCTATTTGGAAAGAGATTGAAATCTCCAATATCTCAGAAATATATAGAATATTTCAGGAATTTATGGAATATTCGATCAAAACCTCTCTTC 8600
bs5 locus TACAAGAATTCAAATTCTATTGGAGTTCTTGTAGGCTATCCTAATCCTAAATTATGTCTATAAAAAGAATGGTTTAATCTTG-AAAAATCATCTTGAATATCCCATAAACCTATTTGGAAAGAGATTGAAATCTCCAATATCTCAGAAATATATAGAATATTTCAGAAATTTATGGAATATTCGATCAAAACCTCTCTTC 8600

Bs5 locus TTGATAATATGAAGATCAAATCTAAGTATTCCTATGTTAGAGACGTTCCTCGAATTACGGAGAAAATAATATGAAGATCAAATCTAAGTATTCCTATGTTAGAGACGTTCCTCGAATTACGGAGAAAATCAAGAGAGAAGAATTAAGAAATTACGGAGAATTACGGAGAAAATCAAGAGAGGAACAGATTTGTACCCGTA 8800
bs5 locus TTGATAATATGAAGATCAAATCTAAGTATTCCTATGTTAGAGACGTTCCTCGAATTACGGAGAAAAT--CAAGAGAGAAGAATTAAGAAATTACGGAGAATTACAGAGAAAATCAAGAGAGGAACAGATTTGTACCCGTA 8800

Bs5 locus TCGTTTATCCGGAAAAAAAAAATCTTATTTTCCTCATATTTTATTTGTGATTACAATTTATTTATGTTCGAATAAAAGTTTATTGGAAACAATTAGGATTGTATAAATTCATGAAAAACACGTTGTAATAATAAGCCATTTTGGGGGAAAGAATTATTGGTAATTTCCTTCCATTCATATCTTATCCCACCATTCTTCTA 9000
bs5 locus TCGTTTATCCGG-AAAAAAAAATCTTATTTTCCTCATATTTTATTTGTGATTACAATTTATTTATGTTCGAATAAAAGTTTATTGGAAACAATTAGGATTGTATAAATTCATGAAAAACACGTTGTAATAATAAGCCATTTTGGGGGAAAGAATTATTGGTAATTTCCTTCCATTCATATCTTATCCCACCATTCTTCTA 9000

Bs5 locus AACTTTCCATGATATTTCCATGTTTTGGAATTATATCATATGGTGTGTGTGTGTATTGTTTCCTAGCCATTTTCAACCTTTTATGATTAGCAACTCCATAGCTTTTAGGGTCACAACTCACAATTATGACATCTTCAACTAAGTGTACTCTTACACAATATGTTTATGATTTTACCGTGTCAAGTCAAACTAAGACACTA 9200
bs5 locus AACTTTCCATGATATTTCCATGTTTTGGAATTATATCATATGGTGTGTGTGTGTATTGTTTCCTAGTCATTTTCAACCTTTTATGATTAGCAACTCCATAGCTTTTAGGGTCACAACTCACAATTATGACATCTTCAACTAAGTGTACTCTTACACAATATGTTTATGATTTTACCGTGTCAAGTCATACTAAGACACTA 9200

Bs5 locus GAAAACGGCGAAACGCTAAAGGGAATGATAGCAAGATCAACACAAAATAAATAAATAGGATACCACGTATCGCGGGAGCTAGCTAGATTGCAGTATCATAATTGTTTTTGGATCAACTGAAATTGTGGTGGAGTGATAAGTATTCCTTCACAACGGATTCCTGGTTTGAGCTTGGGGTATGAAGTTGTTTGTTAGAGAGC 9400
bs5 locus GAAAACGGCGAAACGCTAAAGGGAATGATAGCAAGATCAACACAAAATAAATAAATAGGATACCACGTATCGCGGGAGCTAGCTAGATTGCAGTATCATAATTGTTTTTGGATCAACTGAAATTGTGGTGGAGTGATAAGTATTCCTTCACAACGGATTCCTGGTTTGAGCTTGGGGTATGAAGTTGTTTGTTAGAGAGC 9400

Bs5 locus GTTTCACCCTCCCCTCCATACTCGCCATGTGGGATTCTCCTACACAAATTTAAATTTATATTTAATCGAACTTCAATAACGAATACCGAATACAAAGAGCAAACAAAAAAATTAGATTATTTCAGGAGTTATGACTTTCAATAACGTGCTTTAGAGAACCAATTGCCATTTTTTCTTTTTTTTTTTGACTTTTAAGTCAT 9600
bs5 locus GTTTCACCCTCCCCTCCATACTCGCCATGTGGGATTCTCCTACACAAATTTAAATTTATATTTAATCCAACTTCAATAACGAATACCGAATACAAAGAGCAAACAAAAAAATTAGATTATTTCAGGAGTTATGACTTTCAGTAACGTGCTTTAGAGAACCAATTGCCATTTTTTTTTTTTTTTTTGACTTTTAAGTCAT 9600

Bs5 locus CATTTAATATGAATTGTGAATTAGTAACTAAAGCAAGGAAAGTATTAACAATTCGTTAAACCACCAAAAAAGAAAAAAAACATATGACCAGGTAAATTAATTTAAGTTTTTTTAAAAAAATATATTTAGAGTATCATGTCAACTATCGCCCCCAAAAATATTGGTTTGGTTCGATTAGTGCTTCAATTTGTTCTTTATAA 9800
bs5 locus CATTTAATATGAATTGTGAATTAGTAACTAAAGCAAGGAAAGTATTAACAATTCGTTAAACCACCAAAAAAGAAAAAAAACATATGACCAGGTAAATTAATTTAAGTTTTTTTAAAAAAATATATTTAGAGTATCATGTCAATTATCGCCCCCAAAAATATTGGTTTGGTTCGATTAGTGCTTCAATTTGTTCTTTATAA 9800

Bs5 locus AACTAAATATTATTTGGACTTTAAGTCATACATTCAAATCAATTCAAAACTACATAAAATTTAGTTAGTTTACGTGAAAAATATTTTCCTTCGTTCCAAACGTATTTGTCCTTAATTCAAGTGTTTTTTTAAAACTAGTCAGAGTATCATGTAATTGTTCGCTATTCCCTAAAAAGTGAATTACGTACAGCTTGTAATTA 10000
bs5 locus AACTAAATATTATTTGGACTTTAAGTCATACATTCAAATCAAATCAAAACTACATAAAATTTAGTTAGTTTACGTGAAAAATATTTTCCTTCGTTCCAAACGTATTTGTCCTTAATTCAAGTGTTTTTTTAAAACTAGTCAGAGTATCATGTAATTGTTCACTATTCCCTAAAAAGTGAATTACGTACAGCTTGTAATTA 10000

```
Bs5 locus     AGTCATATATACAAATTATTATTTTACAAGTTGTAGAACTAATGATACAAAAATTTATTTATAATAGAATTAGATAATATATAGTATTTATTACTTGCCCCTTGAAGTTAGCATTATTAGAAAATACTGTACATATATTTTTAAAAATTGACATAACTAGTCTATAAATTTGAATACTATACTAGGTCCAGACGCACAAA 10200
bs5 locus     AGTCATATATACAAATTATTATTTTCCAAGTTGTAGAACTAATGATACAAAAATTTATTTATAATAGAATTAGATAATATATAGTATTTATTACTTGCCCCTTGAAGTTAGCATTATTAGAAAATACTGTACATATATTTTTAAAAATTGACATAACTAGTCTATAAATTTGAATACTATACTAGGTCCAGACGCACAAA 10200

Bs5 locus     TTCCTGATCATACTTAGCAGCTCAAGATATCAGCAGCAGATTCAATAGTGAGTCCTCATCCATCGAGGATATTGTTTCATTTCAGCATTTTAGTATTTCAGTTAGTTCAGTAATTATTTCAGTCGGAGTTAGTTGAGGGTTTGTCTCATCAACTCCATACTCAGAAAGTTAGAGGCTTTCAGACTAGCACGGTCAGTATT 10400
bs5 locus     TTCCTGATCATACTTAGCAGCTCAAGATATCAGCAGCAGATTCAATAGTGAGTCCTCATCCATCGAGGATATTGTTTCATTTCAGCATTTTAGTATTTCAGTTAGTTCAGTAATTATTTCAGTCGGAGTTAGTTGGGGGTTTGTCTCATCAACTCCATACTCAGACAGTTAGAGGCTTTCAGACTAGCACGGTCAGTATT 10400

Bs5 locus     TCAGTTTTCAGATGTTATTTTCAGTATTTTGAGAACCTTATGGCATTAACAGTTATACTTCCGCATATGTTTTCAGTATTATTATTCAGTGCTCACAGCAGGTACCAGTCATGGATTAGCTTGTGATCCTTTGGGGTCGTAAGCACCGTGTGCTGCAGTCTGGAGGCGTTACACACTCTTACCACAAGACCAGAGCCCCG 10600
bs5 locus     TCAGTTTTCAGATGTTATTTTCAGTATTTTGAGAACCTTATGGCATTAACAGTTATACTTCCGCATATGTTTTCAGTATTATTATTCAGTGCTCA--------------CATGGATTAGCTTGTGATCCTTTGGGGTCGTAAGCACCGTGTGCTGCAGTCTGGAGGCGTTACACACTCTTACCACAAGACCAGAGCCCCG 10600

Bs5 locus     AGGACCGGCCTTTATATGCAAGTCAGAAAACAAAAACATTGTACT-ATCTTTCAAGATTTCATTTTCCTTATCAACAACTTTAGTATTCCTGTGACAGTTAACAGACATGCATGCTGTTCCTTTTTGTAAAAGAGAAGCAAAATGGTACATCAGTGAGGTTGTATATAACCTGGGGCTCAATGAGAATAAACTGCAGAAC 10800
bs5 locus     AGGACCGGCCTTTATATGCAAGTCAGAAAACAAAAACATTGTACTTATCTTTCAAGATTTCATTTTCCTTATCAACAACTTTAGTATTCCTGTGACAGTTAACAGACATGCATGCTGTTCCTTTTTGTAAAAGAGAAGCAAAATGGTACATCAGTGAGGTTGTATATAACCTGGGGCTCAATGAGAATAAACTGCAGAAC 10800

Bs5 locus     CCTTTTGTCCTAAAGGGTTGAAGAATTACGCGAACGAATCAAGATACCTACAAGCACGCCTTCGGTTGTATTCTAGCAAGCTTCCATATGTTCGATCCCAGATACCAATGTAAAGGGACTCGTCGTCTGGACTCAGAGATACCCCTGATATCTCACCGAAAAGATCAATTTCCTGACGTTTCTTGTAGTCTGACTCAGTA 11000
bs5 locus     CCTTTTGTCCTAAAGGGTTGAAGAATTACGCGAACGAATCAAGATACCTACAAGCACGCCTTCGGTTGTATTCTAGCAAGCTTCCATATGTTCGATCCCAGATACCAATGTAAAGGGACTCGTCGTCTGGACTCAGAGATACCCCTGATATCTCACCGAAAAGATCAATTTCCTGACGTTTCTTGTAGTCTGACTCAGTA 11000

Bs5 locus     CTGTAAATGTGGACAAAATCTGCAGGTTCAGCCACTACCAGGAACCGTCCATCCGATGAGAAACGGATAGATCGAGCAGCACCGATGTTACCCTTCAGAATGGCCGTAGACGAGGACAAGTTTCTCAAGTCCCAAATTCGACACGTCTTATCTTGATTCCCAGTGGCGAATGTATGGCCATCGGGATGCCACGCACAAGC 11200
bs5 locus     CTGTAAATGTGGACAAAATCTGCAGGTTCAGCCACTACCAGGAACCGTCCATCCGATGAGAAACGGATAGATCGAGCAGCACCGATGTTACCCTTCAGAATGGCAGTAGACGAGGACAAGTTTCTCAAGTCCCAAATTCGACACGTCTTATCTTGATTCCCAGTGGCGAATGTATGGCCATCGGGATGCCACGCACAAGC 11200
Ca_WD40-TC                                                                                          GTTACCCTTCAGAATGGCCGTAGACGAGGACAAGTTTCTCAAGTCCCAAATTCGACACGTCTTATCTTGATTCCCAGTGGCGAATGTATGGCCATCGGGATGCCACGCACAAGC

Bs5 locus     AAAAGAGTAGTCTAAGTGACCAATGATCGACGCCACCGTCTGTTAACAGTAAACAAACTACCATAGATCAGATTTTGGAAGTTCGGCAAATGACGAATTCATATGAAAGCCGAAAAAATATATGGACCACTAGAGTGAGTTCAAACAAATTTCGCTCCCACAAGAAAGATCTATATGAGAAAATACAAGAAACAAACACA 11400
bs5 locus     AAAAGAGTAGTCTAAGTGACCAATGATCGACGCCACCGTCTGTTAACAGTAAACAAACTACCATAGATCAGATTTTGGAAGTTCGGCAAATGGCGAACTCATATGAAAGCCGAAAAAATATATGGACCACTAGAGTGAGTTCAAACAAATTTCGCTCCCACAAGAAAGATCTATATGAGAAAATACAAGAAACAAACACA 11400
Ca_WD40-TC    AAAAGAGTAGTCTAAGTGACCAATGATCGACGCCACCGT-----------------------------------------------------------------------------------------------------------------------------------------------------------------

Bs5 locus     CATAGCGACTCTTTGCAATTTTCTTCGGGAATAATTTTGCCAAACAATCACCTCATCATAAGTGCAGTAACTATATCGACCTAACGTGCTTCAGCATGCCTTCTGACTTTGAAAAAAAA-TAAAAATGATATAACATATTTACATAATCAAAAAAGTTGCACTTCATCTGATTTGTCTGTAGATGTAGTATAAGTGAGGA 11600
bs5 locus     CATAGCGACTCTTTGCAATTTTCTTCGGGAATAATTTTGCCAAACAATCACCTCATCATAAGTGCAGTAACTATATCGACCTAACGTGCTTCAGCATGCCTTCTGACTTTGAAAAAAAAATAAAAATGATATAACATATTTACATAATCAAAAAAGTTGCACTTCATCTGATTTGTCTGTA---------------AGGA 11600
Ca_WD40-TC    --------------------------------------------------------------------------------------------------------------------------------------------------------------------------------------------------------

Bs5 locus     GTATGTATTCATTTCCTCTGAATTGAACGATCAATACTACCATCAGAGTGAAACAAAGGACTAAAGAAGGTAATGGTTAAAAATCTATCATACCTTTCCACTGCGGGAATCAACAAGTAAACCATGTAGATCATCACCAACAACAGTAAAAAGCTTGCAATCTGGGCTCATCGAGGTGTGCTGCATCAATTACCAGTGGA 11800
bs5 locus     GTATGTATTCATTTCCTCTGAATTGAACGATCAATACTACCATCAGAGTGAAACAAAGGACTAAAGAAGGTAATGGTTAAAAATCTATCATACCTTTCCACTGCGGGAATCAACAAGTAAACCATGTAGATCATCACCAACAACAGTAAAAAGCTTGCAATCTGGGCTCATCGAGGTGTGCTGCATCAATTACCAGTGGA 11800
Ca_WD40-TC    ---------------------------------------------------------------------------------------------CTTTCCACTGCGGGAATCAACAAGTAAACCATGTAGATCATCACCAACAACAGTAAAAAGCTTGCAATCTGGGCTCATCGAGGTGTG--------------------

Bs5 locus     ATTAAAAATACATGAGCTCATGAAGGAAGCAGCTTCGAACGGAATTGAAATATCTGTGCACATAAAGCATGTAGACAGGAAGAAGTAATTCTTAATCCAGATTAAAATTGGAGTGAACTGAAACATCTATAATCTTATAATCTCCGAGCAAATGCAATTCAAACTGGATGACGGAAAAGGTCCTGAGTCCGATGAACAAA 12000
bs5 locus     ATAAAAAATACATGAGCTCATGAAGGAAGCAGCTTCGAACGGAATTGAAATATCTGTGCACATAAAGCATGTAGACAGGAAGAAGTAATTCTTAATCCAGATTAAAATTGGAGTGAACTGAAACATCTATAATCTTATAATCTCCGAGCAAATGCAATTCAAACTGGATGACGGAAAAGGTCCTGAGTCCGATGAACAAA 12000
Ca_WD40-TC    --------------------------------------------------------------------------------------------------------------------------------------------------------------------------------------------------------

Bs5 locus     ATCCCGGCTTACTAATAGCAAATCTCATACTATATCTGAAGAAACTAGTGGAAGCTAGATGGAACTTCTTGATAGAGGACAACAGCTTACGTGTTTCCACTATCTACGCGCCAGTTCAGCCCTCTAGGATCAAAAGGCGATCATGTTGGCAATCTACTATATAAGAAGTTGCACCATCAGCCACAGCGATTGTACTCAT 12200
bs5 locus     ATCCCGGCTTACTAATAGCAAATCTCATACTATATCTGAAGAAACTAGTGGAAGCTAGATGGAACTTCTTGATAGAGGACAACAGCTTACGTGTTTCCACTATCTACGCGCCAGTTCAGCCCTCTAGGATCAAAAGGCGATCATGTTGGCAATCTACTATATAAGAAGTTGCACCATCAGCCACAGCGATTGTACTCAT 12200

Bs5 locus     TGAAAACCACTGTGGCACTGATAAATCTAAAACATTCAACCCCCATAAATTGGTTCAATAAGATGAAACGAACGTGAATGACGGAATTTATCTTCAACCGCTTGGCCTTCATCCAACCAACCCATCTAATCTACCACTCAACTCAACCTGAACGTATAAAATTCTTTGATCATCACTCAGATAGCAGGCTATCGCAATAC 12400
bs5 locus     TGAAAACCACTGTGGCACTGATAAATCTAAAACATTCAACCCCCATAAATTGGTTCAATAAGATGAAACGAACGTGAATGACGGAATTTATCTTCAACCGCTTGGCCTTCATCCAACCAACCCATCTAATCTACCACTCAACTCAACCTGAACGTATAAAATTCTTTGATCATCACTCAGATAGCAGGCTATCGCAATAC 12400
Ca_WD40-TC    --------------------------------------------------------------------------------------------------------------------------------------------------------------------------------------------------------

Bs5 locus     AAGGTACTATAAGAAAGAACCTACAAAATAATCACTTCCAGCCTCAATTCAAATCAGAAAGTTGCAGTCCATCTAAAGCCTCGTACTAGAATTTCATTTGATTAAGAAAAGATCATACTAGACATTCATGAAACTACTCTATCCCACTAACACCCAATAGATGACGATGCTGACGCTGATGATCATGATAATCACAATTA 12600
bs5 locus     AAGGTACTATAAGAAAGAACCTACAAAATAATCACTTCCAGCCTCAATTCAAATCAGAAAGTTGCAGTCCATCTAAAGCCTCGTACTAGAATTTCATTTGATTAAGAAAAGATCATACTAGACATTCATGAAACTACTCTATCCCACTAACACCCAATAGATGACGATTCTGACGCTGATGATCATGATAATCACAATTA 12600
Ca_WD40-TC    --------------------------------------------------------------------------------------------------------------------------------------------------------------------------------------------------------

Bs5 locus     CCAGTTTGGAACTTTTCCACAACCTGTCAGTTCATTTGTATGAAAAAGAAAGTATATTTATGCTTCATGGACAACCAAAAACTACATCAGTAACTAATGATAGGAAATCAAAAAAAGCAAAGAAAAGGGAAGAACAAAAGTTGCAGTAAAAGCACATTAAAAACTGACATTCACTGGCCAAGGGAAGCGGAAGTGGTTCA 12800
bs5 locus     CCAGTTTGGAACTTTTCCACAACCTGTCAGTTCATTTGTATGAAAAAGAAAGTATATTTATGCTTCATGGACAACCAAAAACTACATCAGTAACTAATGATAGGAAATCAAAAAAAGCAAAGAAAAGGGAAGAACAAAAGTTGCAGTAAAAGCACATTAAAAACTGACATTCACTGGCCAAGGGAAGCGGAAGTGGTTCA 12800
Ca_WD40-TC    ----------------------------------------------------------------------------------------------------------------------------------------------------------------------ATTCACTGGCCAAGGGAAGCGGAAGTGGTTCA

Bs5 locus     TCTGCTGAAATCTTTCCATGTCGTACACCCTCACACCACAGTCGTTGTTGGCTGCCATAAAACGAGGCCCATAACTGCACAATGTTTACACTTACTTTCATCAGTTGAAGTTTCAAGGTAGCTCAACAACTGATTATGACATTCATACTACCTGACGCTTTCATATATCTCAATGGCATTAGTGATAGCGTTATCCTCAT 13000
bs5 locus     TCTGCTGAAATCTTTCCATGTCGTACACCCTCACACCACAGTCGTTGTTGGCTGCCATAAAACGAGGCCCATAACTGCACAATGTTTACACTTACTTTCATCAGTTGAAGTTTCAAGGTAGCTCAACAACTGATTATGACATTCATACTACCTGACGCTTTCATATATCTCAATGGCATTAGTGATAGCGTTATCCTCAT 13000
Ca_WD40-TC    TCTGCTGAAATCTTTCCATGTCGTACACCCTCACACCACAGTCGTTGTTGGCTGCCATAAAACGAGGCCCATAA-----------------------------------------------------------------------------CTGACGCTTTCATATATCTCAATGGCATTAGTGATAGCGTTATCCTCAT

Bs5 locus     AAGTGGTCCTTGCACAGAAGCTAACCCCCGGCTTGTCCAAATTCTGCAATATTTGAAGCAAAGTTAAGAAAATACCACAAAGAAAGCAAGGATGTATACAGATCAGTAGGACTAAATCATATTAATAGCTCATAATGGTAAGCCAACCATTTAGTAGGGACATAATATCTTCCAGTAAGTTATAGACACCTTAGTTAAAA 13200
bs5 locus     AAGTGGTCCTTGCACAGAAGCTAACCCCCGGCTTGTCCAAATTCTGCAATATTTGAAGCAAAGTTAAGAAAATACCACAAAGAAAGCAAGGATGTATACAGATCAGTAGGACTAAATCATATTAATAGCTCATAATGGTAAGCCAACCATTTAGTAGGGACATAATATCTTCCAGTAAGTTATAGACACCTTAGTTAAAA 13200
Ca_WD40-TC    AAGTGGTCCTTGCACAGAAGCTAACCCCCGGCTTGTCCAAATT-------------------------------------------------------------------------------------------------------------------------------------------------------------

Bs5 locus     ATAAAATGGCGGGAGAGGAACGGAGATTTTCACTTCCGCTACATAAGCTCCAAGCACTAGTACTTCTACTCATATTTAATCAATCATCCTCGTATCATGTTGATTTAGCAGAAAGGAACTTTCTTTTTTTATTTAACTATGTATCTGTTTCCACCAACTAACAGCTCTTTTTCGCTGACCCAAGTCCCAACTAGCCGCAA 13400
bs5 locus     ATAAAATGGCGGGAGAGGAACGGAGATTTTCACTTCCACTACATAAGCTCCAAGCACTAGTACTTCTACTCATATTTAATCAATCATCCTCGTATCATGTTGATTTAGCAGAAAGGAACTTTCTTTTTTTATTTAACTATGTATCTGTTTCCACCAACTAACAGCTCTTTTTCGCTGACCCAAGTCCCAACTAGTCGCAA 13400
Ca_WD40-TC    --------------------------------------------------------------------------------------------------------------------------------------------------------------------------------------------------------

Bs5 locus     ATATTAGCAGTCGTTAAGAGAA-CAGGAAAATACCCAACTGACCTTACAAATGAGTTCTCCTTGGAAGCCCCCAGCAACCACAAAACGGTTCTTCACCGCCATCGTGCTGATTTGGGTCAGTGTAAGACCCTCTAACAAACTTCCTGCATATTTCTGGAGGACCAAAACAGCAGAAAGAATAGGTAAAAACGAAACAGA 13600
bs5 locus     ATATTAGCAGTCGTTAAGAGAAACAGGAAAATACCCAACTGACCTTACAAATGAGTTCTCCTTGGAAGCCCCCAGCAACCACAAAACGGTTCTTCACCGCCATCGTGCTGATTTGGGTCAGTGTAAGACCCTCTAACAAACTTCCTGCATATTTCTGGAGGACCAAAACAGCAGAAAGAATAGGTAAAAACGAAACAGA 13600
Ca_WD40-TC    --------------------------------------------CTTACAAATGAGTTCTCCTTGGAAGCCCCCAGCAACCACAAAACGGTTCTTCACCGCCATCGTGCTGATTTGGGTCAGTGTAAGACCCTCTAACAAACTTCCTGCATATTT---------------------------------------------

Bs5 locus     AATACAAGTAGTTATACACCTAATGAAGAAAAGAGAAAAATGTTAAAAAGGAAAAATTGAGGTGGAACTTCTTTTTCCTGTGTTGCGGCAGGTAACGGTACAGAATACTTCTCTCATACCCACAACAAAGCCAATAAATCAGAGTACTGAGAGGCATACGCACGCAAGACACATTTTTTCAACGCTACATAAAGCATAAT 13800
bs5 locus     AATACAAGTAGTTATACACCTAATGAAGAAAAGAGAAAAATGTTAAAAAGGAAAAATTGAGGTGGAACTTCTTTTTCCTGTGTTGCGGCAGGTAACGGTACAGAATACTTCTCTCATACCCACAACAAAGCCAATAAATCAGAGTACTGAGAGGCATACGCACGCAAGACACATTTTTTCAATGCTACATAAAGCATAAT 13800
Ca_WD40-TC    --------------------------------------------------------------------------------------------------------------------------------------------------------------------------------------------------------
Pr_WD40 R                                                                               5'-TTCTGAGAGGTTGCGGGA →

Bs5 locus     AGGAGGGGAAAGAAGTAAGAGCATAGCAAAAATAAAGTAGGGGACCATCACTAAATACTTTTCAGTTACCTCTGTCGGTACGATACGTCCAGTGAAGTTGACAACTTCTGAGAGGTTGCGGGAAATTGATGACCAATGCATAAGTGAATCGTTTGAAATCAAATACACGTCATGTTTCGAAGTTGCCCACACCAAGTTCC 14000
bs5 locus     AGGAGGGGAAAGAAGTAAGAGCATAGCAAAAATAAAGTAGGGGACCATCACTAAATACTTTTCAGTTACCTCTGTCGGTACGATACGTCCAGTGAAGTTGACAACTTCTGAGAGGTTGCGGGAAATTGATGACCAATGCATAAGTGAATCGTTTGAAATCAAATACACGTCATGTTTCGAAGTTGCCCACACCAAGTTCC 14000
Ca_WD40-TC    ---------------------------------------------------------------------CTCTGTCGGTACGATACGTCCAGTGAAGTTGACAACTTCTGAGAGGTTGCGGGAAATTGATGACCAATGCATAAGTGAATCGTTTGAAATCAAATACACGTCATGTTTCGAAGTTGCCCACACCAAGTTCC

Bs5 locus     TTAGCTGCAAACAAGAGGCACAAGAAGTCACTGAAAGATCTCCACGTAGCAAATTGACCGAAAGCATCACTTGGAAGATAACAACTGACATGAAAAGCACAATAAGGAAATAATTCATTTGGAAACCTAATATTTTTATCTATCACCGGAGGGAAATATAAACGACTTCTTGTTTTTCATTTCAATATGAAAGGGAAAAG 14200
bs5 locus     TTAGCTGCAAACAAGAGGCACAAGAAGTCACTGAAAGATCTCCACGTAGCAAATTGACCGAAAGCATCACTTGGAAGATAACAACTGACATGAAAAGCACAATAAGGAAATAATTCATTTGGAAACCTAATATTTTTATCTATCACCGGAGGGAAATATAAACGACTTCTTGTTTTTCATTTCAATATGAAAGGGAAAAG 14200
Ca_WD40-TC    TTAG----------------------------------------------------------------------------------------------------------------------------------------------------------------------------------------------------

Bs5 locus     AAAAATAAAACACAAGAAAACTGTGTCAGGCACAGAACAGGAAAATCTTTAAGAACATGATCCTTTTCTCTTCAATTCTTTTGTTCTTCATGCTGATTCCCATAGAAGTCGTCCAAAATAAGTTGCTGCTGTAGCATTAAGAATCCAGCATTTAGACATTTGGCAGGTCATCTTTAAGTAGGTGACTAAAACACAAATCT 14400
bs5 locus     AAAAATAAAACACAAGAAAACTGTGTCAGGCACAGAACAGGAAAATCTTTAAGAACATGATCCTTTTCTCTTCAATTCTTTTGTTCTTCATGCTGATTCCCATAGAAGTCGTCCAAAATAAGTTGCTGCTGTAGCATTAAGAATCCAGCATTTAGACATTTGGCAGGTCATCTTTAAGTAGGTGACTAAAACACAAATCT 14400
Ca_WD40-TC    --------------------------------------------------------------------------------------------------------------------------------------------------------------------------------------------------------

Bs5 locus     TTTCAAATTCTAAAGGATAGAAAACACAAACCATCGTTAATCTTAGAGATGGCTAACAGGAAGTGCCACAAGGACACATGTTTTTTTTTACTTAATCAAGTATCAAGATGACAATGATGTTACTTCCACTAAAGAATCTTTCTGACTTTTAAAAGCTAAACAGAACTCAAACTAGAAGAAATACAGGTCCTTCACAGCCA 14600
bs5 locus     TTTCAAATTCTAAAGGATAGAAAACACAAACCATCGTTAATCTTAGAGATGGCTAACAGGAAGTGCCACAAGGACACATGTTTTTTTTTACTTAATCAAGTATCAAGATGACAATGATGTTACTTCCACTAAAGAATCTTTCTGACTTTTAAAAGCTAAATAGAACTCAAACTAGAAGAAATACAGGTCCTTCACAGCCA 14600
Ca_WD40-TC    --------------------------------------------------------------------------------------------------------------------------------------------------------------------------------------------------------

Bs5 locus     CCTGAGATAACTATTTATTTGCATCTAGAAGTTAGAACAGCGTTACACAGCATATACAGAATCTGCAGATTAAATGCAGACCTGAAAATGGAGTATTGTAGGCTTCACGGATCTTGCATTGTAAAAGAATTCGTAGTAGTTGCCACCCCTTTTCCACTTG 14759
bs5 locus     CCTGAGATAACTATTTATTTGCATCTAGAAGTTAGAACAGCGTAACACAGCATATACAGAATCTGCAGATTAAATGCAGACCTGAAAATGGAGTATTGTAGGCTTCACGGATCTTGCATTGTAAAAGAATTCGTAGTAGTTGCCACCCCTTTTCCACTTG 14759
Ca_WD40-TC    ---------------------------------------------------------------------------------CTGAAAATGGAGTATTGTAGGCTTCACGGATCTTGCATTGTAAAAGAATTCGTAGTAGTTGCCACCCCTTTTCCACTTG
Pr_WD40 F                                                                                                                                                ← CGGTGGGAAAAGGTGAAC-5'
```
