# Supplementary material for: The bs5 allele of the susceptibility gene Bs5 of pepper (Capsicum annuum L.) encoding a natural deletion variant of a CYSTM protein conditions resistance to bacterial spot disease caused by Xanthomonas species
Source: Theor Appl Genet. 2023 Mar 21;136(3):64. doi: 10.1007/s00122-023-04340-y (PMC10030403; doi:10.1007/s00122-023-04340-y)
Supplement: Supplementary file 6 — Fig. S6 Tentative consensus sequence of the CYSTM and WD40 genes in pepper. The > 14 kb partial genomic sequence of BAC-50b4 between the flanking genetic markers (M_P6, M_WD40) was used to search homologous sequences in the NCBI “Expressed Sequence Tags” (EST) databases using BLASTN option (http://www.ncbi.nlm.nih.gov/). EST sequences of the five genes were recruited from the Capsicum annuum (taxid:4072) EST database of NCBI (http://www.ncbi.nlm.nih.gov/) using the Bs5 region of BAC-50b4 (see Fig. 1). The individual ESTs (see Table S3) were aligned (using the SeqMan program of the DNA STAR Software; http://www.dnastar.com/), and the consensus sequence generated by the program was taken as the Tentative Consensus (TC; Quackenbush et al. 2000) sequences for Bs5, Bs5-TC (Panel A), for WD40, CaWD40-TC (Panel B) and for Ca_CYSTM2-TC (Panel C). No nucleotide difference was found between the genomic, CYSTM1-TC and CYSTM1-mRNA sequences despite the genomic and EST sequences originated from different pepper cultivars (CaFo, Ca cv. Bukang, Ca cv. Nokkwang and Ca cv. Hang Keun, respectively). Aligning CYSTM1-mRNA and cystm1-mRNA revealed the 6 bp difference between the two sequences [file 122_2023_4340_MOESM6_ESM.pdf]

Fig. S6

A *Bs5-TC* (*Ca_CYSTM1-TC*)

CCCCTTTGACTTTACTCTATAAAAACTTCACAAATATCACCTCTTCACTGTACCCCATTATCTTTCTTTGTGGTTAAGCAAATAC
ACAAAATAAATAAATATAACTCTCCTCTTAGATTAAACTAGTAGATCCATCAACAATGAGTTACTACAATCAACAACAACCTCCT
GTTGGTGTACCTCCACCACAAGGGTATCCACCAGAAGGTTACCCAAAAGATTCATACCCACCACCTGGATATCCACAGCAAGGGT
ACCCTCAACAAGGGTATCCACCACAAGGGTACCCTCCACAGTATGCACCTCAGTATGGTGCACCACCTCCTCAACAACAACATCA
ATCATCTAGTAGTACTGGATTATTGCAAGGATGTTTGGCTGCTCTTTGCTGTTGCTGTCTCTTGGATGCATGCTTTTGATGCTGT
AAATGATCTGTACGCAAAGTGTTGATGACAAAAGATGATTGAAATCCATTATCATAGTCTAGATTATTTTCCTTGAACGTGTTTT
GTCCTTGTTGTCCTGTCATTTATAAATAATTTGATCTTGCTATGGTGTCTATTTGCCAAATTATAGGTTTATGTACAACGTGAGA
GATTGTATTTTATTTTTTATGTTTTGGACCTCAATATGTGAATCAATGCACCTTGATTTGGTTAAA

B *Ca_WD40-TC*

GCACGAGGCACAATAATAGTAATTTATAGTAAAATAAAATACTCAACTCCAACAAATAGGTAGCTATAAAATTAAGCTGAAAAAT
TCCCAAGAAAAAGAAGCTGAAAAATTCCCCAGAAAAAAAGAAAAATTTTCTGTTTGAAAGGTGTGGGAGGGCATGCCTCTTGAAA
GGGTTGACCAATTGGATTACATGGCGGATGACCGGGAGGGGACTGATTCATCTGATGAATTTGAGGGGGGATTATATAATGAGGA
GACAAACCTTGATGAATACGATATGCCAACAAAAGTGACTGATACATCGGCTGCACAAGCAAGAAAAGGGAAGGATATACAAGGT
ATCGCATGGGAGGAATTGAATGTAACGAGGCAAAGCTACAGATTGACAAGGCTTGAACAGTACAGAAATTATGAGAACATCCCTT
TGTCAGGGGAAGCTGTGGATAAGGAATGCAAACAAGTGGAAAAGGGTGGCAACTACTACGAATTCTTTTACAATGCAAGATCCGT
GAAGCCTACAATACTCCATTTTCAGCTAAGGAACTTGGTGTGGGCAACTTCGAAACATGACGTGTATTTGATTTCAAACGATTCA
CTTATGCATTGGTCATCAATTTCCCGCAACCTCTCAGAAGTTGTCAACTTCACTGGACGTATCGTACCGACAGAGAAATATGCAG
GAAGTTTGTTAGAGGGTCTTACACTGACCCAAATCAGCACGATGGCGGTGAAGAACCGTTTTGTGGTTGCTGGGGGCTTCCAAGG
AGAACTCATTTGTAAGAATTTGGACAAGCCGGGGGTTAGCTTCTGTGCAAGGACCACTTATGAGGATAACGCTATCACTAATGCC
ATTGAGATATATGAAAGCGTCAGTTATGGGCCTCGTTTTATGGCAGCCAACAACGACTGTGGTGTGAGGGTGTACGACATGGAAA
GATTTCAGCAGATGAACCACTTCCGCTTCCCTTGGCCAGTGAATCACACCTCGATGAGCCCAGATTGCAAGCTTTTTACTGTTGT
TGGTGATGATCTACATGGTTTACTTGTTGATTCCCGCAGTGGAAAGACGGTGGCGTCGATCATTGGTCACTTAGACTACTCTTTT
GCTTGTGCGTGGCATCCCGATGGCCATACATTCGCCACTGGGAATCAAGATAAGACGTGTCGAATTTGGGACTTGAGAAACTTGT
CCTCGTCTACGGCCATTCTGAAGGGTAAC

C *Ca_CYSTM2-TC*

GGCACGAGAACAAACCCAAATTCCCTCTAACAATGAGTTACTACAATCAACAACAACCCCCTGTTGGTGTACCTCCACCACAAGG
GTATCCACCTGAAGGTTATCCAAAGGATGCTTACCCACCACCAGGGTACCCTCAACAAGGGTATCCACCACAAGGGTACCCTCCA
CAGTATGCACCTCAGTATGGTGCACCACCACCTCAACAACAGCAGCAATCTGGTAGCAGTGGATTTATGGAAGGATGTTTGGCTG
CTCTGTGCTGTTGCTGTCTGTTGGATGCATGCTTTTGATTTGCTGTAAATGACCTGTGCAAGTGTTGATGGCAAGAGATGGATGA
TTCTTCCAATTATATCATAGTCTAGACTTTTTCTTTATGTGTTTTGTCCTTGTTGCCTATAAAGAATTTGATTTTAAGTATGATG
ATGCACCTTTATGTGTTGGAGTATTTTTTGTTTTTGACCTTATTACTATGTGAATGCCACCTTGATTTGATGCTTGGAGTGTCTA
TAACCCAAGCTTTTACTTTTGTGTTGAGAGCCCCGTTGGTTAATCATATTAATAGCTTAGCTTGTTTGTCCAAACTTCTAAAAAG
TAATTTCGGAAGGTAGCGTTACGTGTGTGAATTTCTTCTTGTCAATATTAAAACATTACTTTCTCCGTTTCAAAATAAT
