# Supplementary material for: The bs5 allele of the susceptibility gene Bs5 of pepper (Capsicum annuum L.) encoding a natural deletion variant of a CYSTM protein conditions resistance to bacterial spot disease caused by Xanthomonas species
Source: Theor Appl Genet. 2023 Mar 21;136(3):64. doi: 10.1007/s00122-023-04340-y (PMC10030403; doi:10.1007/s00122-023-04340-y)
Supplement: Supplementary file 7 — Fig. S7 Alignment of Ca_CYSTM2-TC and Bs5-TC. The sequences were taken from Fig. S3, and represents coding, 5′- and 3′-UTR sequences, respectively. Single letter coded amino acids (AA) were deduced from Ca_CYSTM2-TC sequence. Start and Stop codons were underlined. 5′-UTR/3′-UTR and coding regions were highlighted by upper and lower case letters, respectively. Unmatched nucleotides are highlighted with red characters. Nucleotide changes in the coding region which were in the third position of the codon and resulted in no AA change were underlined. The first and second AA in a pair marked deduced AAs from the Ca_CYSTM2-TC and Bs5-TC sequence, respectively. Amino acid similarity highlighted in the paired AA by color: hydrophobic, green; small nonpolar, orange; polar, magenta [file 122_2023_4340_MOESM7_ESM.pdf]

```
Bs5-TC            cctcttagattaaactagtagatccatcaacaATGAGTTACTACAATCAACAACAACCTC  60
Bs5                                                M  S  Y  Y  N  Q  Q  Q  P
Ca_CYSTM2                                          M  S  Y  Y  N  Q  Q  Q  P
Ca_CYSTM2-TC      ggcacgagaacaaacccaaattccctctaacaATGAGTTACTACAATCAACAACAACCCC  60

Bs5-TC            CTGTTGGTGTACCTCCACCACAAGGGTATCCACCAGAAGGTTACCCAAAAGATTCATACC 120
Bs5               P  V  G  V  P  P  P  Q  G  Y  P  P  E  G  Y  P  K  D  S  Y
Ca_CYSTM2         P  V  G  V  P  P  P  Q  G  Y  P  P  E  G  Y  P  K  D  A  Y
Ca_CYSTM2-TC      CTGTTGGTGTACCTCCACCACAAGGGTATCCACCTGAAGGTTATCCAAAGGATGCTTACC 120

Bs5-TC            CACCACCTGGATATCCACAGCAAGGGTACCCTCAACAAGGGTATCCACCACAAGGGTACC 180
Bs5               P  P  P  G  Y  P  Q  Q  G  Y  P  Q  Q  G  Y  P  P  Q  G  Y
Ca_CYSTM2         P  P  P                 G  Y  P  Q  Q  G  Y  P  P  Q  G  Y
Ca_CYSTM2-TC      CACCACC---------------AGGGTACCCTCAACAAGGGTATCCACCACAAGGGTACC 180

Bs5-TC            CTCCACAGTATGCACCTCAGTATGGTGCACCACCTCCTCAACAACAACATCAATCATCTA 240
Bs5               P  P  Q  Y  A  P  Q  Y  G  A  P  P  P  Q  Q  Q  H  Q  S  S
Ca_CYSTM2         P  P  Q  Y  A  P  Q  Y  G  A  P  P  P  Q  Q  Q  Q  Q  S
Ca_CYSTM2-TC      CTCCACAGTATGCACCTCAGTATGGTGCACCACCACCTCAACAACAGCAGCAATC---TG 240

Bs5-TC            GTAGTACTGGATTATTGCAAGGATGTTTGGCTGCTCTTTGCTGTTGCTGTCTCTTGGATG 300
Bs5               S  S  T  G  L  L  Q  G  C  L  A  A  L  C  C  C  C  L  L  D
Ca_CYSTM2         G  S  S  G  F  M  E  G  C  L  A  A  L  C  C  C  C  L  L  D
Ca_CYSTM2-TC      GTAGCAGTGGATTTATGGAAGGATGTTTGGCTGCTCTGTGCTGTTGCTGTCTGTTGGATG 300

Bs5-TC            CATGCTTTtgat--gctgtaaatgatctgtacgcaaagtgttgatgacaaaagatgattg 360
Bs5               A  C  F
Ca_CYSTM2         A  C  F
Ca_CYSTM2-TC      CATGCTTTtgatttgctgtaaatgacctgt--gcaa-gtgttgatggcaagagatggatg 360

Bs5-TC            aaa-t-cca-ttat--catagtctagattattttccttgaacgtgttttgtccttgttgt 420
Ca_CYSTM2-TC      attcttccaattatatcatagtctagact--ttttc-tttatgtgttttgtccttgttgc 420
```
