# Supplementary material for: The bs5 allele of the susceptibility gene Bs5 of pepper (Capsicum annuum L.) encoding a natural deletion variant of a CYSTM protein conditions resistance to bacterial spot disease caused by Xanthomonas species
Source: Theor Appl Genet. 2023 Mar 21;136(3):64. doi: 10.1007/s00122-023-04340-y (PMC10030403; doi:10.1007/s00122-023-04340-y)
Supplement: Supplementary file 9 — Fig. S9. The coding and protein sequence of the CYSTM genes of Nicotiana benthamiana. The coding sequences of Nb_CYSTM1 and Nb_CYSTM2 were retrieved from the SolGene database (https://solgenomics.net/) contigs Niben101Scf02563Ctg059 and Niben101Scf02915Ctg016/Niben101Scf02915Ctg017, respectively. The coding sequence was translated to obtain the deduced protein sequence [file 122_2023_4340_MOESM9_ESM.pdf]

Fig S9.

Nb_CYSTM1-CDS

ATGAGTTACTACAATCAACAACAACCCCCTGTTGGTGTCCCTCCCCCGCAAGGATATCCACCGGAAGGTTACCCAAAGGACGCATACCCACCAC
CGGGGTACCCACCACAGGGATACCCACCACAGGGGTACCCTCAACAAGGTTACCCTCCTCAAGGGTACCCTCCCCAGTATGGTGCTCCACCCCC
TCAACAACAACAGCAACAATCTGGTAGCGCTGGCTTCATGGAAGGATGTTTGGCTGCACTGTGCTGTTGCTGCCTCCTGGATGCATGCTTC

Nb_CYSTM1-Protein

MSYYNQQQPPVGVPPPQGYPPEGYPKDAYPPPGYPPQGYPPQGYPQQGYPPQGYPPQYGAPPPQQQQQQSGSAGFMEGCLAALCCCCLLDACF

Nb_CYSTM2-CDS

ATGAGTTACTACAATCAACAACAACCCCCTGTTGGTGTCCCTCCCCCGCAAGGATATCCACCGGAAGGTTACCCAAAGGATGCATACCCACCAC
CAGGTTATCCACCACAGGGGTACCCACCACAGGGGTACCCTCAACAAGGTTACCCTCCTCAGTATGGTGCTCCACCCCCTCAACAACAACAGCA
ACAATCTGGTAGCGCTGGCTTCATGGAAGGATGTTTGGCTGCACTGTGCTGTTGCTGTCTCCTGGATGCATGCTTC

Nb_CYSTM2-Protein

MSYYNQQQPPVGVPPPQGYPPEGYPKDAYPPPGYPPQGYPPQGYPQQGYPPQYGAPPPQQQQQQSGSAGFMEGCLAALCCCCLLDACF
