# Supplementary material for: The bs5 allele of the susceptibility gene Bs5 of pepper (Capsicum annuum L.) encoding a natural deletion variant of a CYSTM protein conditions resistance to bacterial spot disease caused by Xanthomonas species
Source: Theor Appl Genet. 2023 Mar 21;136(3):64. doi: 10.1007/s00122-023-04340-y (PMC10030403; doi:10.1007/s00122-023-04340-y)
Supplement: Supplementary file 10 — Supplementary file10 (DOCX 15 kb) [file 122_2023_4340_MOESM10_ESM.docx]

**Table S1** Primers used in this study

Primer name Primer sequence Amplification Marker name

size (bp)

*Pr_CaCY F* 5’- GTGGCTCATGCTGTGGATTTCT 366 *M_CaCY*
*Pr_CaCY R* 5’- CCAGGAGTGCAGGGGTAGGTTA

*Pr_AF244121-A F* 5’- CACCTCCTCGCCAATCCTTCTG 237 *M_AF244121*
*Pr_AF244121-A R* 5’- TTGAAAGTTCTTTCCATGACCAACC

*Pr_WD40 F* 5’- CAAGTGGAAAAGGGTGGC 854 *M_WD40*
*Pr_WD40 R* 5’- TTCTGAGAGGTTGCGGGA

*Pr_P6 F* 5’- TTTCGTGAGTATTATTCCTTTTTA 726 *M_P6*
*Pr_P6 R* 5’- CGCTGCTTTTTCGCTATGT

*Pr_50b4-OP F* 5’- ACCAACTAGAATCCAAAT 225 *M_50b4-OP*
*Pr_50b4-OP R* 5’- TGAACTTAAAGATGCTGA

*Pr_50b4-40 F* 5’- ATGATTTCTATGATGGCTAG 173 *M_50b4-40*
*Pr_50b4-40 R* 5’- GTTGGAAGTATTGGGTTAA

*Pr_279g12-OP F* 5’- GCTGGTCTATCTTGATCCTTCA 396 *M_279g12-OP*
*Pr_279g12-OP R* 5’- ATGTCCCTCCCTGTCATTCTAT

*Pr_279g12-40 F* 5’- TGGGACTAATAAGGAAAGAA 145 *M_279g12-40*
*Pr_279g12-40 R* 5’- GAAGTGATGAAAGTGGGTTG

*Pr_326d1-OP F* 5’- TTTGCTGTAAATATGGGTC 405 *M_326d1-OP*
*Pr_326d1-OP R* 5’- GTCGAGCATAGACATTGAGATA

*Pr_326d1-40 F* 5’- GGGGAACCTTGGAGTAAC 127 *M_326d1-40*
*Pr_326d1-40 R* 5’- CATAAGGGTGTATGGTGT

*Pr_632h6-OP F* 5’- TCAACAAAGGCAGCAGAATG 227 *M_632h6-OP*
*Pr_632h6-OP R* 5’- TTCTGCTCTTTTCCCCTGAA

*Pr_632h6-40 F* 5’- TTGCCAGAAGTTGTCCTATT 143 *M_632h6-40*
*Pr_632h6-40 R* 5’- ATTGTCTTGTTGTGCGTTAT

*Pr_877a8-40 F* 5’- ATGTCAAGAATCACAACCGTA 258 *M_877a8-40*
*Pr_877a8-40 R* 5’- GTAAGATGGCCGATTAATATG

*Pr_1045d3-OP F* 5’- cgaaacattgatgtggtgat 152 *M_1045d3-OP*
*Pr_1045d3-OP R* 5’- ttgttggcgtcttctttgat

*Pr_1191b6-OP F* 5’- ACGAGCAAATAGAAGGCAATG 147 *M_1191b6-OP*
*Pr_1191b6-OP R* 5’- CACCCTCTACAAGAAACTCT

*Pr_1191b6-40 F* 5’- AAAACTGGGTTAATGTTGGG 149 *M_1191b6-40*
*Pr_1191b6-40 R* 5’- CGTGGCGGCTGTATTGTCTC

*Pr_1248g5-OP F* 5’- ACGAGCTTGAGATACTGA 326 *M_1248g5-OP*
*Pr_1248g5-OP R* 5’- CTCTTGGGAAAGGTCATA

*Pr_1248g5-40 F* 5’- GTCTTACATGCCCCAAAT 207 *M_1248g5-40*
*Pr_1248g5-40 R* 5’- CATCACGAGCACTACCTG

*Pr_Bs5g F3* 5’- TCTGCAGTGCACAATCTAAACAAATGACCA 3047
*Pr_Bs5g R3* 5’- ACTGCAGTTGCACCAGGAGTTCATCCG

*Pr_Bs5c F7* 5’- GCTAGCATGAGTTACTACAATCAACAA 291
*Pr_Bs5c R7* 5’- cacgtgtcaAAAGCATGCATCCAAG

*Pr_Bs5c F7* 5’- GCTAGCATGAGTTACTACAATCAACAA 279
*Pr_bs5c R7T* 5’- cacgtgTCAAAAGCATGCATCACA

*Pr_bs5g F1* 5’- CTGCTCTTTGCTGTTGCT 183/189 *M_bs5g*
*Pr_bs5g R1* 5’- GGCAAATAGACACCATAGC

*Pr_Neo F* 5’- AGTGACAACGTCGAGCACAG 176 *M_Neo*

*Pr_Neo R* 5’- AGACAATCGGCTGCTCTGAT

*Pr_Hyg F* 5’- TCGCTAAACTCCCCAATGTC 163 *M_Hyg*

*Pr_Hyg R* 5’- GCGAAGAATCTCGTGCTTTC

*Pr_ipt F* 5’- ATTTTCGGTCCAACTTGCAC 278 *M_ipt*
*Pr_ipt R* 5’- CCTCCCTCAAGAATAAGCCC
