# Supplementary material for: The bs5 allele of the susceptibility gene Bs5 of pepper (Capsicum annuum L.) encoding a natural deletion variant of a CYSTM protein conditions resistance to bacterial spot disease caused by Xanthomonas species
Source: Theor Appl Genet. 2023 Mar 21;136(3):64. doi: 10.1007/s00122-023-04340-y (PMC10030403; doi:10.1007/s00122-023-04340-y)
Supplement: Supplementary file 11 — Supplementary file11 (DOCX 77 kb) [file 122_2023_4340_MOESM11_ESM.docx]

**Table S2.** Results of the complementation experiments

| **Code of transgenic plants** | **Presence of the genes in the transformants** | | | | | **Presence of flowers on the transformants** | **Mature seeds** | **Sensitivity to Xe78** |
| --- | --- | --- | --- | --- | --- | --- | --- | --- |
|  | ***Ipt*** | ***Hyg^R^*** | ***Neo^R^*** | ***Ca_CYSTM1 (Bs5)*** | ***Ca_cystm1 (bs5)*** |  |  |  |
| **TR116.2.56** | **+** | **+** | **-** | **+** | **+** | **+** | **-** | **S** |
| **TR116.4.77** | **+** | **+** | **-** | **+** | **+** | **+** | **-** | **S** |
| **TR116.4.97** | **+** | **+** | **-** | **+** | **+** | **+** | **-** | **S** |
| **TR116.4.103** | **+** | **+** | **-** | **+** | **+** | **+** | **-** | **S** |
| **TR116.4.154** | **+** | **+** | **-** | **+** | **+** | **+** | **-** | **S** |
| **TR121.2.19** | **+** | **-** | **+** | **+** | **+** | **+** | **-** | **S** |
| **TR116.2.82** | **+** | **-** | **+** | **+** | **+** | **+** | **+** | **S** |
| **C1; C2; C3** | **+** | **+** | **-** | **-** | **+** | **+** | **+** | **R** |

**
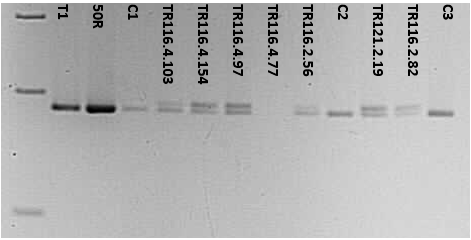
**

**Table SI 1**. *C. annuum* cv. *Global* plants were inoculated by *A. tumefeciens ShooterGRif^R^* (pCambia-1303- Ca_CYSTM1), *A. tumefeciens ShooterGRif^R^* (pCambia-1303-Ca_CYSTM1) and as a control by *A. tumefeciens ShooterGRif^R^* (pCambia-1303) strains according to the transformation protocol described by Mihalka et al. (2003). Five *Hyg^R^* (TR116.2.56; TR116.2.82; TR116.4.77; TR116.4.97; TR116.4.103; TR116.4.154), two *Neo^R^* (TR121.2.19, TR121.2.82) and 3 control *Hyg^R^* (C1; C2; C3) independent shoot transformants were selected on agar matrix. The functional and structural map of pCambia-1303 can be found at the following homepage: http//:www.cambia.org/daisy/cambia/585. Abbreviations: *ipt,* isopentenyl transferase, *Hyg^R^*, hygromycin phosphotransferase resistance gene, *Neo^R^*, neomycin phosphotransferase resistance gene, Xe78, *Xanthomonas euvesicatoria* strain 78. For genotyping *ipt, Hyg*^R^, *Neo*^R^, Bs5/bs5 gene, M Ipt, M Hyg, M Neo and M_bs5g markers, were used respectively (Table S3).
