# Supplementary material for: The bs5 allele of the susceptibility gene Bs5 of pepper (Capsicum annuum L.) encoding a natural deletion variant of a CYSTM protein conditions resistance to bacterial spot disease caused by Xanthomonas species
Source: Theor Appl Genet. 2023 Mar 21;136(3):64. doi: 10.1007/s00122-023-04340-y (PMC10030403; doi:10.1007/s00122-023-04340-y)
Supplement: Supplementary file 12 — Supplementary file12 (DOCX 15 kb) [file 122_2023_4340_MOESM12_ESM.docx]

**Table S3** Identification numbers for EST sequneces used in this study

**A** Identification numbers (ID no.) for EST sequences of *Bs5* gene recovered from the EST databank of *Capsicum* (taxid:4071) of NCBI (<http://www.ncbi.nlm.nih.gov/>)

EST ID no. GenBank Acc.no. *Capsicum annuum* Tissue expressed

cultivars (cv.)

1. BJ02011A07 CO906966.1 *Nokkwang*  red pepper fruit pericarp

2. BJ02024B09 CO907872.1 *Nokkwang* red pepper fruit pericarp

3. BJ02028F07 CO908250.1 *Nokkwang* red pepper fruit pericarp

4. BJ03007G10 CO910563.1 *Nokkwang*  red pepper fruit pericarp

5. BJ03012D10 CO910903.1 *Nokkwang* red pepper fruit pericarp

6. KS01021E10 BM060865.1 *Bukang* 8 weeks after germination

7. KS01042G03 BM062434.1 *Bukang* 8 weeks after germination

8. KS01066G10 BM064421.1 *Bukang* leaf inoculated with *X. campestris*

9. KS08004A01 BM067385.1 *Hang Keun* 10 weeks after germination

10. KS11011F01 CA520379.1 data not found

11. KS15009H05 GD064264.1 *Bukang* Fruit, mature green pericarp seeds

12. KS17039H08 GD079197.1 *Bukang* Fruit, mature red ripe pericarp

13. KS18012C05 GD082583.1 *Bukang* Seeds

14. KS18020C12 GD082583.1 *Bukang* Seeds

15. KS18027H11 GD084043.1 *Bukang* Seeds

16. KS19002B03 GD087557.1 *Bukang* Flower bud

17. KS22027E06 GD107514.1 *Bukang* Peduncle

18. KS22055G10 GD110165.1 *Bukang* Peduncle

19. KS23038F06 GD114576.1 *Bukang* Callus

20. KS26050B10 GD134228.1 *Bukang* rbcS-silenced leaves

21. KS26053E04 GD134525.1 *Bukang* rbcS-silenced leaves

**B.**  Identification numbers (ID no.) for EST sequences of *CaCYSTM2* gene recovered from the EST databank of *Capsicum* (taxid:4071) of NCBI (<http://www.ncbi.nlm.nih.gov/>)

EST ID no. GenBank Acc.no. *Capsicum annuum* Tissue expressed

cultivars (cv.)

1. KS07012E08 BM066264.1 *Hang Keun* flower bud

2. KS20020F12 GD095184.1 *Bukang* open flower

**C.** Identification numbers (ID no.) for EST sequences of *CaWD40* gene recovered from the EST databank of *Capsicum* (taxid:4071) of NCBI (<http://www.ncbi.nlm.nih.gov/>

EST ID no. GenBank Acc.no. *Capsicum annuum* Tissue expressed

cultivars (cv.)

1. BM062317.1 KS01041B10 *Bukang*  leaf inoculated with *X. campestris*

2. CA516649.1 KS09061E01 data not found

3. CO776107.1 JH02006C12 *Nokkwang* red ripe fruit pericarp

4. CO906634.1 BJ02005B02 *Nokkwang* red pepper fruit pericarp

5. CO907408.1 BJ02017C11 *Nokkwang* red pepper fruit pericarp

6. GD126168.1 KS25026E10 *Bukang*  Germination stage 18 day

7. GD127299.1 KS25039D09 *Bukang*  Germination stage 18 day

8. GD127880.1 KS25045I15 *Bukang*  Germination stage 18 day
